# Development and usability testing of an online support tool to identify models and frameworks to inform implementation

**DOI:** 10.1186/s12911-024-02580-6

**Published:** 2024-06-27

**Authors:** Lisa Strifler, Christine Fahim, Michael P. Hillmer, Jan M. Barnsley, Sharon E. Straus

**Affiliations:** 1 Knowledge Translation Program, Li Ka Shing Knowledge Institute, St. Michael’s Hospital, Unity Health Toronto, Toronto, ON Canada; 2 Institute of Health Policy Management & Evaluation, University of Toronto, Toronto, ON Canada; 3 Ontario Ministry of Health, Toronto, ON Canada; 4 Department of Medicine, University of Toronto, Toronto, ON Canada

**Keywords:** Implementation, Knowledge translation, Model, Framework, Support tool

## Abstract

**Background:**

Theories, models and frameworks (TMFs) are useful when implementing, evaluating and sustaining healthcare evidence-based interventions. Yet it can be challenging to identify an appropriate TMF for an implementation project. We developed and tested the usability of an online tool to help individuals who are doing or supporting implementation practice activities to identify appropriate models and/or frameworks to inform their work.

**Methods:**

We used methods guided by models and evidence on implementation science and user-centered design. Phases of tool development included applying findings from a scoping review of TMFs and interviews with 24 researchers/implementers on barriers and facilitators to identifying and selecting TMFs. Based on interview findings, we categorized the TMFs by aim, stage of implementation, and target level of change to inform the tool’s algorithm. We then conducted interviews with 10 end-users to test the usability of the prototype tool and administered the System Usability Scale (SUS). Usability issues were addressed and incorporated into the tool.

**Results:**

We developed *Find TMF*, an online tool consisting of 3–4 questions about the user’s implementation project. The tool’s algorithm matches key characteristics of the user’s project (aim, stage, target change level) with characteristics of different TMFs and presents a list of candidate models/frameworks. Ten individuals from Canada or Australia participated in usability testing (mean SUS score 84.5, standard deviation 11.4). Overall, participants found the tool to be simple, easy to use and visually appealing with a useful output of candidate models/frameworks to consider for an implementation project. Users wanted additional instruction and guidance on what to expect from the tool and how to use the information in the output table. Tool improvements included incorporating an overview figure outlining the tool steps and output, displaying the tool questions on a single page, and clarifying the available functions of the results page, including adding direct links to the glossary and to complementary tools.

**Conclusions:**

*Find TMF* is an easy-to-use online tool that may benefit individuals who support implementation practice activities by making the vast number of models and frameworks more accessible, while also supporting a consistent approach to identifying and selecting relevant TMFs.

**Supplementary Information:**

The online version contains supplementary material available at 10.1186/s12911-024-02580-6.

## Background

Theories, models and frameworks (TMFs) offer valuable information about implementation processes and outcomes to researchers and implementers (i.e., individuals who are doing or supporting implementation practice activities such as planning, evaluating and sustaining evidence-based healthcare interventions). For example, models (also referred to as “process models”) offer practical guidance by specifying key steps in the process of implementing evidence into practice; evaluation frameworks are useful to evaluate implementation quality and outcomes; determinant frameworks describe the types of factors that affect implementation outcomes; and theories are helpful to understand or explain why and how different factors influence implementation [1]. Yet, researchers and implementers continue to face challenges when identifying and selecting relevant TMFs from the over one hundred available options [2]. This challenge is reflected in explicit calls for more informed and transparent use of TMFs prior to and throughout implementation research and practice activities [1, 3, 4] and includes a need to facilitate the process of identifying and selecting an appropriate TMF for a given implementation project [5, 6].

Existing guides and tools offer support to those who are looking to identify and select an appropriate implementation TMF. For example, Lynch et al. [7] published a practical guide for use by clinical researchers and clinicians with key questions to consider when selecting a TMF. Tabak et al. [8] published an inventory of 61 TMFs based on a narrative review that was subsequently used by Rabin et al. to inform an interactive web tool called Dissemination and Implementation (D&I) Models [9]. D&I Models (https://dissemination-implementation.org/) is designed for researchers and implementers to consult when selecting and applying TMFs and identifying measures to assess key TMF constructs. It is important to note that this web tool does not distinguish implementation models from frameworks and theories, and the differences between these terms are significant and warrant distinction based on their intended purpose or aim (as per the TMF definitions above) [1]. Terminology in the implementation science literature is also inconsistent, as some process models (e.g., the Knowledge-to-Action [or KTA] Framework) are referred to as frameworks or theories and vice versa, adding to the challenge faced by individuals when trying to identify an appropriate TMF to inform their specific implementation project.

To assist researchers and implementers with selecting among two or more relevant TMFs, Birken et al. [5] developed an Implementation Theory Comparison and Selection Tool (T-CaST) with four categories of selection criteria: applicability, usability, testability and acceptability (https://impsci.tracs.unc.edu/tcast/). More recently, Moullin et al. [4] published a worksheet and a checklist to facilitate their recommendations for selection and comprehensive application of one or more TMFs across an implementation project. Both tools have been published in open access implementation-specific journals. When using these tools, users must develop their own condensed list of relevant TMFs that are specific to their implementation project. This limitation has been identified by the tool developers [5]. As such, these tools are not intended to support users in sifting through the multitude of available TMFs.

To address these gaps, we aimed to rigorously develop a comprehensive and user-centered online tool to help individuals who are doing or supporting implementation practice activities to identify appropriate TMFs to inform their work. Our first objective was to design and develop the online support tool prototype by applying the findings from two previously published studies that we conducted to inform this work – a comprehensive scoping review of TMFs [10] and individual interviews with researchers and implementers to understand the barriers and facilitators to identifying and selecting relevant TMFs [11]. Our second objective was to test the usability of the prototype tool to determine end-user experience with the tool and develop recommendations for its improvement.

## Methods

**Fig. 1:**
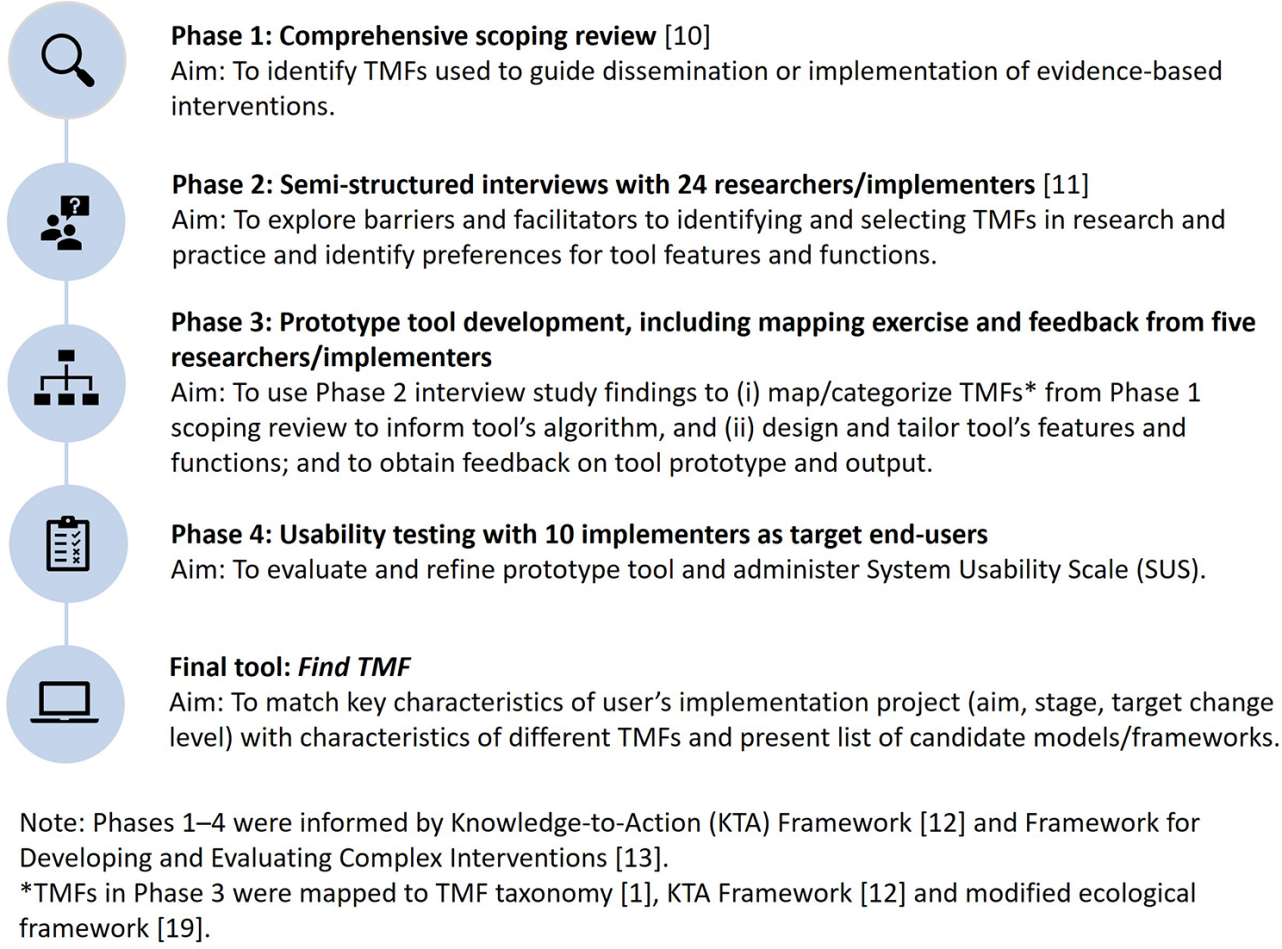
Phases of tool development

The approach for tool development and usability testing (Fig. 1) followed rigorous methods guided by models and evidence on implementation science and user-centered design. Specifically, our overarching multi-phase approach was informed by two models, the KTA Framework [12] and the Framework for Developing and Evaluating Complex Interventions [13]. These models and methods have been used in combination to develop other implementation/knowledge translation support tools [14].

### Phase 1: Comprehensive scoping review to identify TMFs

Our approach to tool development began by conducting a scoping review to identify TMFs used to guide dissemination or implementation of evidence-based interventions. We followed the scoping review methods by Arksey and O’Malley [15] and the Joanna Briggs Institute [16]. Our scoping review search included 305 TMF names, and we screened 4,598 records. Overall, 596 studies reporting on the use of 159 TMFs were included in our review. The results of our phase 1 scoping review were published elsewhere [10].

### Phase 2: Semi-structured interviews to understand barriers and facilitators

To understand barriers and facilitators to identifying and selecting TMFs in research and practice, we conducted 24 semi-structured interviews with researchers and implementers from Canada, the United States and Australia. Participants identified barriers related to: characteristics of the individual or team supporting an implementation project, program or initiative (e.g., knowledge about and experience or training in TMFs), characteristics of the TMF (e.g., clear and concise language, good fit with the implementation context), and characteristics of the implementation project, program or initiative (e.g., consideration of the purpose/problem or goal and intended outcome, implementation stage, and target level of change). The results of our phase 2 interview study were published elsewhere [11].

### Phase 3: Prototype tool development, including mapping exercise to categorize TMFs and feedback from researchers/implementers

Following a user-centered approach, we used the results from our phase 2 interview study, together with a comprehensive list of TMFs identified through our phase 1 scoping review, to inform and tailor the tool’s content and functions. To inform the tool’s algorithm, we conducted a mapping exercise to categorize a comprehensive list of TMFs obtained from the following two sources: 305 TMFs were identified in our scoping review search process and 159 TMFs were then included from the 596 studies included in our scoping review [10], and 36 TMFs were identified in an updated and related scoping review by Esmail et al. [17]. After combining the sources and removing duplicates, 323 TMFs were included in the mapping exercise. We obtained the original full text publication for each TMF, and two investigators (LS, CF) independently mapped them in duplicate according to three criteria: intended purpose (theory, model, framework), scope (stage of implementation) and target level of change (individual, organizational, system). These criteria were informed by our phase 2 interview findings and are described in more detail below.

First, we categorized the list of TMFs by their intended purpose using Nilsen’s taxonomy [1] of five approaches: process models, determinant frameworks, evaluation frameworks, classic theories and implementation theories. This taxonomy is useful for understanding the different overarching aims or purposes that TMFs serve in implementation science and practice. We then categorized the TMFs by scope or implementation stage using the KTA Framework [12]. This frequently cited [18] process model is comprehensive and includes all stages of implementation: identifying the evidence gap and adapting the evidence to the local context; assessing barriers/facilitators to evidence use and selecting, tailoring and implementing interventions; monitoring evidence use and evaluating implementation outcomes; and sustaining evidence use. Further, the KTA Framework is used by national and international health agencies (e.g., Canadian Institutes of Health Research, World Health Organization) to frame their implementation/knowledge translation activities and was reported as being the most used TMF by participants (67%) in our phase 2 interview study. Finally, we categorized the TMFs by intended or target level of change using a modified ecological framework perspective [19] consisting of individuals (e.g., changing behaviour of patients or healthcare professionals), the organization (e.g., change within a healthcare team or hospital) and the system (e.g., change within a network of hospitals or at the community or policy level). The detailed mapping criteria can be found in the Supplemental File. A pilot exercise of 15 randomly selected TMFs (5%) was completed in duplicate and compared to ensure full agreement on mapping before proceeding with the full exercise. Disagreements in mapping were resolved through discussion, or by a third investigator (SES) as needed.

With support from a web developer who has experience in implementation tool design, we used the criteria and results of the mapping exercise to directly inform the tool algorithm and questions and developed a prototype online tool. Prior to usability testing, we sought informal feedback on the prototype tool’s design features, functions and output from five researchers/implementers within our circle of contacts at the Knowledge Translation Program (St. Michael’s Hospital, Unity Health Toronto) who expressed interest in the tool. These individuals were provided with access to the online prototype tool and asked to share their written feedback and suggestions. Comments and suggestions were either incorporated into the tool or logged for further exploration during our usability study.

### Phase 4: Usability testing with end-users

Once developed, we conducted a usability study to evaluate and refine the prototype tool. Through individual semi-structured interviews with end-users of the tool, we explored how users interacted with the prototype tool (including actions, verbalizations and issues) and how well the tool met their needs [20]. We followed the Consolidated Criteria for Reporting Qualitative Research checklist for reporting this study [21]. Research ethics approval was obtained from the Unity Health Toronto Research Ethics Board (#20–038) and the University of Toronto Research Ethics Board (#42891). Verbal informed consent was obtained and recorded at the start of each interview.

#### Participant selection and recruitment

Eligibility criteria included individuals involved in or who support the implementation of evidence-informed practices, policies or programs in healthcare environments (e.g., hospitals, community settings) in Canada or internationally, as end-users of the tool. The target sample size was 5–8 end-users, as this number is recommended to uncover a majority (approx. 85%) of usability problems [22]. Interviews ended once our target sample size was met, and no new issues were raised.

To recruit participants, we included an ad for our usability study in the Knowledge Translation Canada weekly e-newsletter for 4 weeks in March and April 2023. Knowledge Translation Canada (www.ktcanada.org) is a network of Canadian experts on the uptake of evidence in practice, and its e-newsletter has over 2,000 national and international subscribers, the majority (approx. 75%) are from Canada (personal communication with MS, Knowledge Translation Canada). We sent the study information sheet to eligible participants who responded to the ad and if interested, we asked them to contact us to schedule an interview. In addition, we emailed the study sheet to participants from our phase 2 interview study who provided consent to be contacted for future research and used snowball sampling with our usability study participants.

#### Data collection and analysis

Interviews were conducted by one investigator (LS) using a video conferencing platform (Zoom Video Communications Inc) to view the participant’s computer screen as they completed two tasks using the prototype tool. Each task included a written description of a healthcare scenario and instructions to identify an appropriate TMF to inform the scenario. We developed the scenario and instructions based on findings from our phase 2 interview study and investigator expert opinion (SES). Using a think-aloud approach, we asked participants to say what they were thinking, looking at and trying to do as they interacted with the tool to complete the task and review the output [23]. The tasks were presented in a random order, by generating a random sequence using the Microsoft Excel RAND function. Each task had a time limit of 10 min. Following the completion of both tasks, we administered the System Usability Scale (SUS) [24]. The SUS consists of ten questions on intention to use, ease of use and confidence to use and five response options ranging from 1-strongly disagree to 5-strongly agree. Individual SUS scores range from 0 to 100; a score greater than 70 is considered “acceptable” usability [25]. This scale is a widely used and reliable measure of subjective usability and has been validated with individuals rating the usability of websites [25]. The interview guide also contained probing questions on tool content, format, navigation and purpose to serve as prompts when needed. The study scenarios and interview guide can be found in the Supplemental File.

The audio/video recordings were transcribed and analyzed using content analysis. All data were coded by a single investigator (LS), with the first two transcripts reviewed by a second investigator (SES) to ensure consistency of the findings. The data were summarized descriptively for problems encountered while navigating the tool to complete the tasks, general feedback on the tool (e.g., content, format/navigation, purpose) and suggestions for improvement [20]. We calculated total task time to complete each task, as well as individual SUS scores as composite measures of overall usability [24]. The findings of our content analysis were incorporated into the tool by our web developer on an ongoing basis throughout usability testing [20].

## Results

### Phase 3: Prototype tool development, including mapping exercise to categorize TMFs and feedback from researchers/implementers

#### Mapping exercise

We excluded 113 of 323 TMFs during the mapping exercise: one was a duplicate, 33 were not implementation focused, 42 did not map to Nilsen’s taxonomy (e.g., implementation strategy, taxonomy, theoretically rooted tool/algorithm) and 37, which initially mapped to Nilsen’s taxonomy, did not map to the KTA Framework or were too restrictive and not useful for understanding barriers or implementation (e.g., highly specialized topic or single barrier, process model for a specific implementation strategy). The mapping results for the remaining 210 TMFs are presented in Table 1.

**Table 1:** Results of mapping exercise for 210 TMFs

Mapping criteria		Model	Evaluation framework	Determinant framework	Theory	Total
Stage of implementation	Select and/or adapt knowledge	53	2	17	4	61
	Identify barriers and facilitators	40	9	82	57	138
	Select, tailor and implement strategies	70	6	41	40	124
	Monitor knowledge use and/or evaluate outcomes	59	11	23	6	76
	Sustain knowledge use	29	4	17	2	41
Target level of change	Individual	65	14	74	64	172
	Organizational	74	11	61	17	126
	System	56	9	45	11	96
Total		86	16	97	71	210

Almost half (46%) of 210 TMFs met the criteria for a determinant framework, followed by model (41%), theory (34%) and evaluation framework (8%). Fifty-four TMFs mapped to more than one TMF category; the most common category combinations were theory and determinant framework (*n* = 24) and model and determinant framework (*n* = 22). The most common stages of implementation addressed by the TMFs were identify barriers and facilitators (66%) and select, tailor and implement strategies (59%), while the least common stage was sustain knowledge use (20%) (Table 1). Eighty-two of 210 TMFs mapped to one KTA stage, 62 to two stages, 38 to three stages, 20 to four stages and 8 to all 5 stages of the KTA Framework. Most TMFs mapped to individual level change (82%) and fewer to system level change (46%). Further, 19 TMFs mapped to both individual and organizational level, 13 to organizational and system, 4 to individual and system, and 74 to all three levels of change.

#### Prototype tool

The online tool, which can be freely accessed through the tool website, is intended for use by individuals who are involved in planning or supporting implementation practice activities and are looking to identify an appropriate TMF for their specific implementation project. The prototype tool algorithm included process models (*n* = 86), determinant frameworks (*n* = 97) and evaluation frameworks (*n* = 16). Theories (*n* = 45) were excluded from the prototype due to their unique aims and characteristics, with plans to include them in the future – for example, the user would need to consider the components of their intervention and the desired outcomes, as well as the underlying assumptions that link them, against theories suggested by the tool [3]. The tool consisted of 3 to 4 questions about the user’s specific implementation project. The first question asked the user to describe the purpose or goal of their implementation project in an open textbox. This first question was intended for the user to clarify the purpose or goal of their project before proceeding with the tool. The subsequent three questions in the tool corresponded to our mapping exercise. For example, the focus was on whether the user was looking for a process model to describe or guide the implementation process; a determinant framework to understand or explain factors related to implementation and/or sustainability; or an evaluation framework to understand or explain implementation quality/outcomes and/or sustainability. If looking for a process model, the user was asked to select all relevant stages of implementation for their project: select and/or adapt evidence; identify barriers and facilitators and/or select, tailor and implement strategies; monitor evidence use and/or evaluate outcomes; and sustain evidence use. The user was also asked to identify the target change level (individual, organizational, and/or system level change) for their initiative. Depending on the user’s answers to these questions, the tool’s algorithm matched key characteristics of their implementation project with characteristics of different models or frameworks and presented a list of options that could be applicable to their project. Additional information was provided in the output table, for each potentially relevant model or framework, to help the user narrow down their list of candidate options depending on their needs. This information included: the original citation, a link to the PubMed abstract (when available), the number of citations (updated in real-time using a Dimensions Badge, available at https://www.dimensions.ai/), whether the model or framework has a figure, and the original discipline or condition. The user had the option to sort the results alphabetically and export (i.e., in .csv or .xlsx formats) and save their list for further consideration.

Five researchers/implementers (4 from Canada, 1 from the United States) provided written feedback on the tool prototype and output during the development phase. Reviewers commented that they liked the look and simplicity of the tool, found the tool questions to be helpful, liked having the option to click on the question mark icon next to each tool question for more detailed information including examples and references, and found the output useful. However, two reviewers commented that depending on how the questions were answered, the output could produce many frameworks in the results, which could be challenging to sift through. Reviewers’ suggestions for improvement that were incorporated into the tool prior to usability testing included: improving accessibility by incorporating more plain language and definitions; and offering guidance to users for selecting among the models or frameworks in the output by including information on available complementary tools. Other reviewer suggestions – to consider offering a guided tutorial of the tool for first time users and/or including an overview figure of the steps for completing the tool and offering users the ability to view the entire database of included TMFs – were noted for further exploration in our phase 4 usability study.

### Phase 4: Usability testing with end-users

Ten individuals from Canada or Australia participated in our usability study across four rounds of testing, with improvements incorporated into the tool after each round. Interviews took place in 2023 from May to July (*n* = 4, round 1), October to November (*n* = 2, round 2; *n* = 2, round 3) and December (*n* = 2, round 4). Participants had a range of experience supporting implementation practice activities (range 3–30 years.) Characteristics of the study participants are listed in Table 2.

**Table 2:** Characteristics of usability study participants (*n* = 10)

**Country**	***n***
Canada	9
Australia	1
**Years of experience supporting implementation practice activities**	*n*
1–5 years	5
6–10 years	2
>10 years	3

The mean time to task completion was 10:52 (1:23) (minutes: seconds [standard deviation, SD]) for the first scenario (A or B) and 7:30 (1:59) (minutes: seconds [SD]) for the second scenario (B or A). The mean SUS score was 84.5 (SD 11.4).

#### Tool purpose and content

Participants across all rounds of testing showed an understanding of the tool’s purpose and described advantages to using *Find TMF* in practice (Table 3). Participants also commented on the credibility of the tool and liked that it was “informed by both the research literature as well as user preferences and ideas” (ID1, round 1). Most participants (*n* = 7) said they would like to use the tool frequently. Further, some participants (*n* = 4) offered to disseminate the tool within their network of colleagues or through their health organization’s channels.

**Table 3:** Usability interview excerpts reflective of tool purpose and content

*“The advantages are numerous because it allows you to sift through the myriad of theories, models and frameworks that are out there to select one or more that will be most applicable, most useful to the question that you’re trying to address in your implementation project.” (ID2, round 1)*
*“I think a tool like this can definitely help broaden people’s perspectives as to what is available for their use and what they can explore with regards to how they frame their implementation project or initiative. I think this will be really helpful.” (ID5, round 2)*
*“I could see using it for most, if not all, of my projects.” (ID7, round 3)*
*“It just kind of goes back to who is going to use this tool and do you need a bit more around here’s why we need theories, models and frameworks. This is what it will help you do. But if people have already found themselves here, maybe that’s not needed.” (ID6, round 2)*
*“What should I be doing now? Because it feels like it’s super structured and then you get all these results and it’s like now what?” (ID1, round 1)*
*“I think the tool explains the purpose. You come up with these results of TMFs, but what do I do with these TMFs? I think the tool is really great at credibility around those TMFs, like showing their evidence but…I’m thinking about implementation practitioners who are doing implementation activities and…one of the things they would highlight…would be…where it showed, oh great I can go look up some articles and see examples of how this is used or more description about the TMF to help them determine, of that list of whatever I got, to be able to go oh I’ve got it [narrowed] down, or to bring it back to their team members to say this is what we’re looking at.” (ID9, round 4)*

**Fig. 2:**
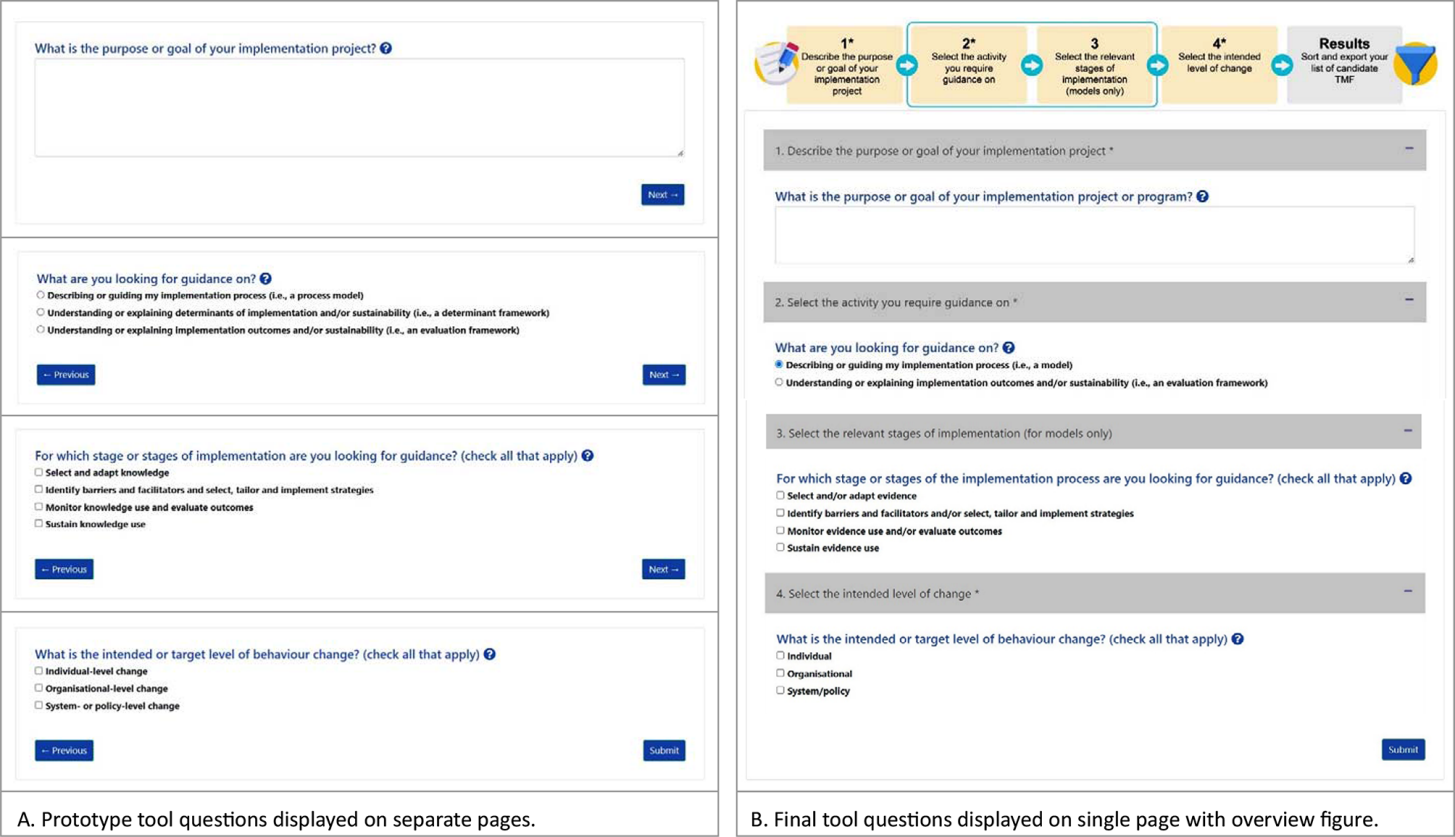
Screenshots of tool questions at start (**A**) and end (**B**) of usability study

Table 4 provides a list of the feedback provided during usability testing and the corresponding changes that were made to the prototype tool after each round. Overall, participants commented that the tool’s concepts and questions were relevant and clear, and found the additional information and examples provided in the help icon instructions for each question helpful. A few participants (*n* = 3) suggested clarifying the tool’s audience on the homepage and felt that users would require some level of knowledge of TMFs and why they are helpful. All participants in round 1 (*n* = 4) suggested tool improvements to give new users a better idea of what to expect when completing the tool for the first time. Prior to round 2, a graphic designer created an overview figure showing the number of steps and output for inclusion on the tool’s homepage, questions page (Fig. 2B) and results page. One participant in round 3 suggested including a case study to walk first-time users through the tool.

**Table 4:** Feedback from usability testing (*n* = 10) and revisions to prototype tool

Feedback	Revisions
Clarify target audience and level of knowledge required to use tool	After round 2: –Clarified target audience on homepage. –Added link from homepage to glossary to define: “theory”, “model”, “framework” and “implementation practice activities”.
Give users better idea of what to expect when completing tool for first time	After round 1: –Added link from homepage to information on how to use tool. –Created overview figure showing number of steps and output for inclusion on homepage, questions page and results page. After round 3: –Incorporated numbering and used colour to better link tool questions to overview figure. After round 4: –Included case study with scenario and screenshots of tool.
Show user’s response to question 1 (What is the purpose or goal of your implementation project?) on screen while completing tool	After round 1: –Changed presentation of tool questions from one question per page to accordion-style format with all questions presented on single page. –Added summary box to results page to display user’s answers to tool questions.
Clarify that users may need to complete tool more than once, depending on needs	After round 1: –Revised help icon instructions for question 2 (What are you looking for guidance on?). –Clarified instructions on how to use tool page.
Too many TMFs in results table	After round 2: –Revised tool algorithm and question 2 (What are you looking for guidance on?) to restrict tool to models and/or evaluation frameworks, with option to download list of theories and determinant frameworks. –Added three new columns to output table with TMF year, whether TMF is model or framework and target change level.
Provide more guidance to users on what to do with tool output and table functionality	After round 2: –Clarified instructions on results page and added direct links to glossary and to complementary tools.

Participants agreed that it was important that the tool output offered users more than one TMF to choose from. One participant was not aware that so many TMFs existed: “I think it’s really impressive that it’s providing so many options” (ID4, round 1). However, all participants in rounds 1 and 2 (*n* = 6) commented that too many models or frameworks in the results could be overwhelming and suggested providing more guidance to users on what to do with the output (Table 4). To address these concerns prior to round 3, we revised the tool algorithm and question 2 (What are you looking for guidance on?) to restrict the tool to 99 models and/or evaluation frameworks by removing 66 determinant frameworks (which ranged from broad to very specific in focus, and most of which (*n* = 52) included individual level change). We also clarified the instructions on the results page and added a direct link to complementary tools including T-CaST. Participants found it helpful to review the information provided in the output table for each model or framework, including being able to access articles containing a description of the TMF and examples of its use and knowing the original discipline or condition. Further, round 3 and 4 participants found that three new columns in the output table (year, whether the TMF is a model or framework, level of change) further assisted them in narrowing down their results (Table 4*)*. Other suggestions to include a brief summary of the TMF and a visual of the TMF figure were beyond the scope of the current study (e.g., due to copyright issues).

#### Tool format and function

Participants in all rounds of testing found the tool to be visually appealing and easy to use and liked the simplicity of having 3–4 questions to answer (Table 5). Regarding the format of the tool questions, most participants in round 1 (*n* = 3) commented that it would be helpful to have their response to question 1 (What is the purpose or goal of your implementation project?) visible on the screen while completing the tool (Table 4). In addition, when answering tool question 2 (What are you looking for guidance on?), two participants in round 1 struggled with not being allowed to select more than one response option for process model or framework. A round 3 participant suggested to better link the tool questions to the overview figure at the top of the page through numbering and use of colour. Screenshots of the tool questions in the prototype tool at the start of usability testing versus the final version of the tool at the end of usability testing are displayed in Fig. 2.

**Table 5:** Usability interview excerpts reflective of tool format and function

*“I think if you are able to help someone really clarify their aim or goal and then hold that, if you can transfer that to these next few pages to then be able to answer these [tool] questions, it can have this thought process be a little bit more succinct.” (ID3, round 1)*
*“I like how it looks. Visually, it has a very clean, organized and neat look and I like the simplicity of it… I think really for anyone that has a basic understanding of just implementing and sort of conducting an evaluation, I think this could be intuitive for them, especially with the ‘more information’ prompts you have available too. Because that definitely provides more guidance as to how to answer the questions.” (ID5, round 2)*
*“I found the questions very helpful, very lay-friendly enough that you know, I found them easy to answer. I like these little grey dropdown menus. So it doesn’t feel overwhelming when I’m looking at just a stack of things to answer. I can go step by step which I found very nice…and I do like that when you finish it and hit submit that that little results step on the top right-hand side lights up so you know where you are.” (ID7, round 3)*
*“I think in general it was good that there weren’t a lot of questions. Made it quite streamlined I guess that you could just click a few things and then you get the options shown to you” (ID4, round 1)*
*“It is super simple, easy to understand. I like that there’s not a lot of information that can confuse people. They have three main questions they have to answer and … they already have this very basic information. So that’s great to facilitate the adaptation of the tool. People can really adapt to it.” (ID8, round 3)*
*“That’s useful that I can sort through these by discipline or whether or not it has a figure. Things that would just help. It’s so anxiety provoking at the beginning of implementation, trying to even start thinking about theories, models and frameworks. So this is super helpful to narrow things down and give you different ways to start all of those considerations. And it’s nice to know when things have been developed and how long they’ve been in use.” (ID10, round 4)*

Participants across all rounds of testing liked the format and functionality of the output table, including links to access the PubMed abstract and citations and the ability to export and save their results. However, some participants in rounds 1 (*n* = 1) and 2 (*n* = 2) struggled to navigate the sort and export features within the table (Table 4*)*. Screenshots of the *Find TMF* tool results page can be found in the Supplemental File. One participant in round 3 suggested including a blank worksheet document for users to download and fill in with the TMFs they are considering and want to explore further; this function will be considered in future iterations of the tool.

## Discussion

The implementation (or knowledge translation) field is heterogeneous as it addresses implementation problems in a variety of contexts. *Find TMF* attempts to distinguish the characteristics of models and frameworks for implementation, and how they could be used to address different types of user needs. Our multi-phase approach to tool development included a scoping review to identify TMFs, followed by semi-structured interviews with 24 researchers and implementers to explore barriers to identifying and selecting TMFs in research and practice. To develop the prototype tool and inform the algorithm, we mapped in duplicate 210 TMFs identified in our scoping review, according to three criteria (their purpose/aim, stage of implementation, and target level of change) based on factors identified in our interview study. The most mapped categories for purpose and stage of implementation are consistent with the findings from a recent scoping review by Wang et al. [26] that assessed the usability, applicability and testability of 143 TMFs using criteria adapted from Nilsen’s TMF taxonomy [1] and Birken et al.’s T-CaST [5]. Similarly, the least frequently mapped stage of implementation was sustain knowledge use, the omission of which could limit the applicability of a TMF for implementers who are doing or supporting long-term work to sustain improvements.

Iterative changes were made to the prototype tool based on feedback from implementers. The tool’s usability was improved by incorporating an overview figure outlining the tool steps and output, displaying the tool’s questions on a single page, and clarifying instructions on the functions of the output table. While the mean SUS score of 84.5 (SD 11.4) was within the “good” to “excellent” usability range [25], future acceptance and use of *Find TMF* by end-users will be influenced by factors such as perceived ease of use, perceived usefulness and attitudes toward using the tool [27, 28].

**Fig. 3:**
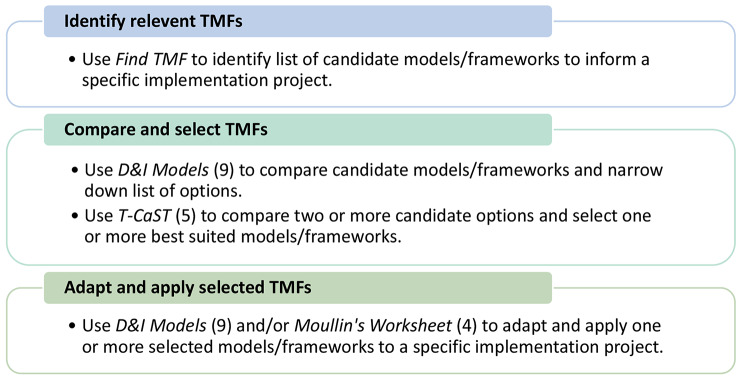
Complementary support tools to identify, select and apply TMFs

To demonstrate the value-add of our tool, it will also be important to compare the use of *Find TMF* with other existing support tools to explore how they might best complement each other. For example, Fig. 3 shows how *Find TMF* could be used alongside D&I Models [9], T-CaST [5] and/or Moullin’s worksheet [4] to identify, select and apply a TMF. More recently, Porat-Dahlerbruch et al. [29] published a scoping review protocol outlining plans to identify attributes and criteria specific to TMF selection for implementation strategy design, and to use the results to inform the development of an online tool for researchers. The scope of their proposed tool could therefore be less comprehensive than *Find TMF*, with a focus on providing users with TMFs to guide a single stage of the KTA Framework for implementation strategy design, whereas *Find TMF* includes models and frameworks that address each and all implementation stages and is targeted to implementers.

### Limitations

To inform our tool algorithm, we coded the TMFs using a frequently cited taxonomy [1], and categorized and structured the TMFs in a linear approach using the well-respected KTA Framework [12] as broad stages of implementation. We acknowledge that the use of these approaches may have biased our results as some TMFs did not fit into these categories and were therefore excluded. As such, it is important to note that the use of a different taxonomy and/or process model for our mapping exercise could have resulted in a different algorithm in the tool. Further, while we chose to code each TMF according to the purpose or aims explicitly mentioned in the original publication for that TMF, we acknowledge that some TMFs are being applied in the literature in new ways. For example, the Theoretical Domains Framework was originally developed and validated as a determinant framework to identify factors that affect implementation [30] but has since been used in the literature for both planning and evaluation purposes [1]. Future updates to the TMF coding in the tool should take into consideration any new applications in the literature, and will require conducting a separate literature review for each TMF. Similarly, while it was not feasible to assess and report on the limitations to the different TMFs, implementers would benefit from reviews of the quality and applicability of each existing TMF to assist with the selection process.

Next, depending on how a user answers the questions in the tool, the output of models or frameworks could provide numerous options, leading the user to gravitate toward the TMFs that are familiar without considering the other options being presented. As such, determinant frameworks were removed from the tool during usability testing as participants commented that the output was too large to sift through, especially for those targeting individual level change. However, we view the tool’s output as a strength, in that it aims to provide users with a broad range of TMFs for consideration, which could in turn increase the possibility of using a model and/or framework that is more applicable to their specific implementation project. Given that *Find TMF* can serve as a pre-cursor to T-CaST, future revisions to our tool’s content should consider including T-CaST criteria such as whether the TMF provides step-by-step guidance for application, what are the included constructs, who is the target audience, and links to empirical support for the TMF, to help users narrow down their list. T-CaST criteria could also inform the inclusion of additional information in the output table to accommodate the future inclusion of determinant frameworks and theories in the tool. In the meantime, a list of theories and determinant frameworks will be available for users to download in the tool.

A major strength of our work is the use of quantitative and qualitative methods guided by models and evidence on implementation science and user-centered design to develop and test the usability of the tool. Further, to our knowledge our mapping work, which informed the tool’s algorithm, resulted in the first inventory of TMFs that are categorized by both a TMF taxonomy and a full-spectrum process model. The mapping results will be useful to both researchers and implementers as it provides a basis to identify and compare relevant TMFs by intended purpose or aim and stages of implementation. Given that high-quality implementation tools are crucial to optimize implementation practice [31], we hope that *Find TMF* will contribute to advancing both implementation science, through an increased awareness and use of available models and frameworks, and implementation practice, through improved implementation planning and outcomes [6, 32].

The findings and positive feedback from our usability study offer promising support for the use of *Find TMF* in practice. *Find TMF* will be made publicly available and disseminated within Canada and internationally, as we continue to refine and update the tool’s content and algorithm. Depending on the resources available to maintain the tool, such approaches should include incorporating data and feedback from end-users (e.g., making the tool open access so that users can provide input and suggest additions), updating the scoping review and TMF database, and exploring the value and benefit of a living review of TMFs to inform implementation.

## Conclusion

*Find TMF* is a simple and easy-to-use online support tool that aims to make the multitude of available models and frameworks more accessible and support a consistent approach to identifying and selecting relevant TMFs for an implementation project.

### Electronic supplementary material

Below is the link to the electronic supplementary material.

Supplementary Material 1

## Data Availability

Data is provided within the manuscript or supplemental file.

## Abbreviations

D&I: dissemination and implementation
KTA: Knowledge–to–Action
SUS: System Usability Scale
T-: CaST Implementation Theory Comparison and Selection Tool
TMF: theory, model and/or framework
