# Supplementary material for: Development and usability testing of an online support tool to identify models and frameworks to inform implementation
Source: BMC Med Inform Decis Mak. 2024 Jun 27;24:182. doi: 10.1186/s12911-024-02580-6 (PMC11209996; doi:10.1186/s12911-024-02580-6)
Supplement: Supplementary file 1 — Supplementary Material 1 [file 12911_2024_2580_MOESM1_ESM.pdf]

SUPPLEMENTAL FILE: Development and usability testing of an online support tool to identify models and frameworks to inform implementation

Appendix 1. Mapping criteria 2
Appendix 2. Consolidated criteria for reporting qualitative studies (COREQ) checklist 7
Appendix 3. Usability study scenarios with instructions, and interview guide 10
Appendix 4. Results of mapping exercise for 210 TMFs 15
Appendix 5. References for 210 TMFs from mapping exercise 28
Appendix 6. Screenshots of final tool homepage and results page 38

Appendix 1. Mapping criteria

The purpose of this exercise is to map a list of TMFs to (1) Nilsen's taxonomy and (2) the Knowledge-to-Action (KTA) Framework and categorize them by (3) level of behaviour change (individual, organizational, system). The results will be used to inform the content of a support tool to help individuals who are doing or supporting implementation practice activities to identify an appropriate TMF to inform their work.

(1) Nilsen's Taxonomy (Nilsen, 2015):

Category^	Description[1]	Similarities and differences[1]
**Process models**	- Specify steps/stages/phases in process of translating research into practice, including implementation and use of research<br>- Aim to describe and/or guide process of translating research into practice<br>- An action model is type of process model that provides practical guidance in planning and execution of implementation endeavors and/or implementation strategies to facilitate implementation<br>- Note that terminology is inconsistent, as some models are referred to as frameworks and vice versa.	Process models vs. frameworks:<br>- While many process models mention relevance of addressing B/Fs to translating research into practice (e.g., KTA), these models do not identify or systematically structure specific determinants associated with implementation success like frameworks do<br>- Process models recognize temporal sequence of implementation endeavors (although actual process is not necessarily sequential), whereas determinant frameworks do not explicitly take a process perspective of implementation since determinants typically relate to implementation as whole
**Determinant and evaluation frameworks**	- Many frameworks developed by synthesizing results from empirical studies of B/Fs to implementation success or constructed based on synthesis of constructs related to behaviour change found in behaviour change theories<br>- ***Determinant frameworks*** describe/specify types/classes/ domains of determinants and individual determinants, which act as B/Fs and enablers (IV)	Frameworks vs. process models:<br>- Determinant frameworks may be *used* to help guide implementation practice (i.e., function as action models), because they identify potential B/Fs that might be important to address when undertaking implementation endeavor. However, most determinant frameworks provide limited "how-to" support for carrying out implementation endeavors since determinants usually are too generic to provide

[1] Source: Nilsen. Making sense of implementation theories, models and frameworks. Implement Sci 2015;10:53.

	that influence implementation outcomes (DV)<br>- Aim to understand and/or explain influences on implementation outcomes, e.g., predicting outcomes or interpreting outcomes retrospectively<br>- May specify relationships between some types of determinants<br>- ***Evaluation frameworks*** specify aspects of implementation that could be evaluated to determine implementation success	sufficient detail for guiding implementation process<br>- Evaluation framework could also be process model if it contains process pieces<br><u>Frameworks vs. theories:</u><br>- A lot of theory has been used to identify determinants of behaviour, but the resulting framework does not address how change takes place or any causal mechanisms (e.g., Theoretical Domains Framework, a framework, versus Health Action Process Approach, a theory).
**Classic and implementation theories**	- Aim to understand and/or explain aspects of implementation by addressing how change takes place (i.e., the causal mechanisms) without ambitions to actually bring about change.<br>- <u>Expanded definition</u>: "'A set of concepts and/or statements with specification of how phenomena relate to each other. Theory provides an organizing description of a system that accounts for what is known, and explains and predicts phenomena'.[2] …[must] include a set of concepts that describe, explain and predict phenomena including the relationships between the concepts".[3]<br>- ***Classical theories*** originate from fields external to implementation science (e.g., psychology, sociology and organizational theory).	<u>Theories vs. process models:</u><br>- Theories may be *used* to guide implementation practice (i.e., function as action models), because they identify potential B/F that might be important to address when undertaking implementation endeavor. However, theories typically describe change mechanisms and explain how change occurs without ambitions to bring about change (i.e., the process).<br><u>Theories vs. frameworks:</u><br>- See <u>Frameworks vs. theories</u> above<br>- Theories may be *used* for evaluation because they describe aspects that might be important to evaluate.

[2] Hobbs et al. Behaviour change theories across psychology, sociology, anthropology and economics. A systematic review. Psychol Health 2011;26(Supp 2):6-72.
[3] Colquhoun et al. A systematic review of the use of theory in randomized controlled trials of audit and feedback. Implement Sci. 2013;8:66.

	- ***Implementation theories*** have been developed by implementation researchers, from scratch or by adapting existing theories and concepts.	
**None of the above**	- Approaches that do not meet any of Nilsen's definitions of TMF should go here (a critique of his taxonomy).	E.g., taxonomy, checklist, principles, tools

B/Fs = barriers and facilitators

Note: ^Code TMF according to intended aim as described in its original publication.

(2) KTA Framework (Graham et al., 2006):

**KTA Stage**	**Notes[4]:**<br>**Does the TMF aim to describe or understand/explain aspect of, or specify how to…**
**Define the evidence to practice gap**<br>- Identify problem<br>- Determine the know/do gap<br>- Identify, review, select knowledge	- Identify the problem?<br>- Assess the current level of an activity?<br>- Assess what "best practices" are or what the 'know' part of this stage entails?<br>- Measure the "gap" between evidence and practice or policy making?<br>- Conduct a needs assessment (e.g., using admin dataset or chart audit) to determine size and nature of the gap?<br>- Identify and appraise relevant research that could help solve the gap?<br>- Actively involve relevant stakeholders in identifying and prioritizing the knowledge gap?
**Adapt knowledge to local context**	- Adapt the knowledge to the local practice environment/context? (e.g., guideline adaptation)
**Assess barriers/ facilitators (B/Fs) to knowledge use**	- Assess B/Fs to knowledge use? (e.g., Delphi procedure, focus groups, interviews, questionnaires, statistical analyses on types of data)<br>- Map identified B/Fs to behaviour change techniques and/or intervention components? (approach may include qualitative participatory methods, PDSA cycles, or theory-based approaches such as Michie's intervention mapping)
**Select, tailor, implement interventions to promote knowledge use**	- Select/incorporate intervention components to develop a KT intervention? (approach may include intervention mapping, marketing, precede/proceed, quality cycle, change management, organizational development, community development, and health technology assessment)

[4] Sources: table adapted from intersectionality & KT project work at Unity Health Toronto (personal communication with Kasperavicius); Straus et al. Knowledge Translation in Health Care, 2nd Ed. 2013; CIHR, http://www.cihr-irsc.gc.ca/e/40618.html; Straus et al. Monitoring use of knowledge and evaluating outcomes. CMHJ, 2010;182(2):E94-8.

	- Tailor the selected KT intervention components to the specific B/Fs to which they map? (e.g., using exploratory or theory-based approach)<br>- Implement a KT intervention that has been designed?
**Monitor knowledge use**	- Assess uptake of the knowledge to determine how and to what extent the knowledge is used by decision-makers?<br>o Conceptual knowledge use: changes in levels of knowledge or understanding or in attitudes (e.g., intentions to change)<br>o Instrumental knowledge use: changes in behavior or practice (e.g., adherence to recommendations)<br>o Strategic (persuasive) knowledge use: use of knowledge for political change and to influence policy (e.g., use in policy-related documents)<br>- Note: TMF requires feedback and evaluation (e.g., process evaluation) to meet this step
**Evaluate outcomes**	- Determine the impact of use of knowledge on outcomes specific to health, provider and/or system?<br>o Patient: impact on patients of using or applying the knowledge (e.g., health status, HR-QOL, satisfaction with care)<br>o Provider: impact on providers of using or applying the knowledge (e.g., satisfaction with practice, time taken to do new practice)<br>o System: impact on health system of using or applying the knowledge (e.g., length of stay, cost, waiting times)
**Sustain knowledge use**	- Plan for sustainability (continued implementation of KT interventions over time), spread (e.g. expanded to new setting or context) and/or scaling up (e.g. expanded to reach greater proportion of eligible population) of knowledge use?<br>- Maintain change, adapt and sustain ongoing knowledge use?

(3) Level of change:[5]

**Level**	**Definition**	**Example**
**Individual**	Changing behaviour of a single person, or a group of individuals that are bound by common characteristics but don't necessarily work collectively	This level deals with the individual/person level (e.g., psychological TMF)
**Organizational**	Change within the confines of a single organization. Individual change is part of organizational change, but what makes	This level deals with groups of individuals – it could be a small team or a full organization such as a hospital or social institution

[5] Sources: Moore et al. Public Health Action Model for Cancer Survivorship. Am J Prev Med. 2015;49(6 Suppl 5):S470-6; Intersectionality & KT project work at Unity Health Toronto (personal communication with Kasperavicius).

	organizational change differ from individual change is the concept of collective behaviour/action (e.g., collective commitment to change, collective values and interests, etc.)	(e.g., sociologically-based TMF and those based in organizational behaviour)
**System/policy**	"Large-system transformations in health care are interventions aimed at coordinated, systemwide change affecting multiple organizations and care providers, with the goal of significant improvements in the efficiency of health care delivery, the quality of patient care, and population-level patient outcomes."[6]	This level deals with groups larger than the organization – how do different hospitals (including multiple hospitals in a large network) or different provinces or countries interact within themselves and beyond themselves<br>*Add a note in the comments column when the TMF applies to policy-level

[6] Best et al. Large-System Transformation in Health Care: A Realist Review. Milbank Q. 2012; 90(3):421-56.

Appendix 2. Consolidated criteria for reporting qualitative studies (COREQ) checklist

Item	Description
**Domain 1: Research team and reflexivity**	
1. Interviewer/facilitator	Lisa Strifler
2. Credentials	BSc, MSc, PhD candidate
3. Occupation	PhD student
4. Gender	Female
5. Experience and training	Strifler is a PhD candidate who conducted this research as part of her PhD thesis project. She has training in health services research and methods and received formal training in qualitative research methodology during her graduate studies.
6. Relationship established	Email communication was exchanged between Strifler and the participant to determine eligibility and schedule an interview.
7. Participant knowledge of the interviewer	Participants were aware of the purpose and rationale of the study, that it was being conducted as part of Strifler's PhD thesis project, and the project funding source.
8. Interviewer characteristics	Strifler disclosed her role as a PhD candidate at the University of Toronto, a graduate trainee with the Knowledge Translation Program at St. Michael's Hospital in Toronto, Canada, and primary researcher on this project.
**Domain 2: study design**	
9. Methodological orientation and theory	Content analysis
10. Sampling	Convenience and snowball sampling were used
11. Method of approach	Participants were recruited via an ad in the Knowledge Translation Canada e-newsletter (which ran for 4 weeks in March and April 2023) or by snowball sampling with our usability study participants. Participants in our previous study, who provided consent to be contacted for participation in future research, were also invited to participate in the usability study via personalized email.
12. Sample size	10 participants. A target sample size of 5-8 participants was expected to provide sufficient information to answer the research question through semi-structured interviews and was considered a feasible range given the available resources.
13. Non-participation	13 individuals either responded to the ad in the e-newsletter (n=11, of which 5 did not book an interview) or were referred by a usability study participant (n=2). 7 eligible participants in our previous study were sent an email invitation, of which 4 did not respond and 1 was not reached due to an undeliverable email address. Participants were recruited until no new usability issues were identified; therefore, not all of the 24 participants in our previous study were contacted/invited to participate.
14. Setting of data collection	Interviews were conducted online using a video conferencing platform. Participants participated from their preferred location.

15. Presence of non-participants	No non-participants were present during the interviews.
16. Description of sample	See Table 2.<br>All participants were from Canada (Ontario, Alberta or British Columbia) except for 1 participant who was from Australia. Participants worked in a variety of healthcare environments and had a range of experience supporting implementation activities in healthcare environments.
17. Interview guide	See Supplemental File Appendix 3.<br>A semi-structured interview guide was prepared and revised as needed throughout data collection. The interview guide was reviewed by a clinician/knowledge translation expert who had experience with qualitative research and implementation science and practice.
18. Repeat interviews	Repeat interviews were not conducted.
19. Audio/visual recording	Interviews were audio and video-recorded using an online video conferencing platform and transcribed verbatim.
20. Field notes	Strifler took notes during and immediately following each interview and referred to these notes during data analysis and interpretation.
21. Duration	Interviews lasted 30 to 60 minutes.
22. Data saturation	Participants were recruited until no new usability issues were identified.
23. Transcripts returned	Individual transcripts were not returned to participants for comment.
**Domain 3: analysis and findings**	
24. Number of data coders	Data were inductively coded by a single investigator. The first 2 coded transcripts (a subset of 20%) were reviewed by a second investigator.
25. Description of the coding tree	A description of the coding tree has not been provided but will be made available upon request.
26. Derivation of themes	Themes were derived from the data.
27. Software	NVivo 12 Plus qualitative data analysis software (QSR International, Cambridge, MA) was used to organize and code the transcripts.
28. Participant checking	Participant checking was not performed, as changes were made to the tool iteratively, throughout usability testing.
29. Quotations presented	See Tables 3 and 5.<br>Direct quotes from participants were presented in tables to support the study findings. Each quotation was identified using participant ID numbers.
30. Data and findings consistent	The data and findings are consistent.
31. Clarity of major themes	See Table 4.<br>Major issues/suggestions for improvement included additional instruction and guidance on what to expect from the tool and

	how to use the information in the output table. Tool improvements included: (1) incorporating an overview figure outlining the tool steps and output, (2) displaying the tool questions on a single page, and (3) clarifying the available functions of the results page, including adding direct links to the glossary and to complementary tools.
32. Clarity of minor themes	See text in Results section.<br>Issues/suggestions for improvement and corresponding changes made to the tool were further described within the text in the Results under sections for (1) tool purpose and content and (2) tool format and function.

Appendix 3. Usability study scenarios with instructions, and interview guide

Scenario A:

Please take a moment to read the scenario aloud. Once you are familiar with the scenario, please take a few minutes to reflect on what you might want to consider, and complete the task described below using the support tool. As you are doing so, please "think aloud" and provide any feedback including saying what you are thinking, what you are looking at and what you are trying to do.

You will have up to 10 minutes to complete the task. Do you have any questions?

SCENARIO
Background: Adults aged 65 and older account for a high percentage of acute hospital stays. Evidence suggests that early and consistent mobilisation of older adults admitted to hospital can decrease acute care length of stay, increase functional status and increase rates of discharge to home. Yet, rates of mobilisation in patients admitted to hospitals remain low. To address this gap, the Mobilisation of Vulnerable Elders (MOVE) initiative aims to promote early and consistent mobilisation practices for older adults admitted to hospitals. The three key components of this evidence-based program are to: mobilise patients at least three times a day; use progressive, scaled mobilisation; and, assess mobility within 24 hours of the decision to admit.<br>Task: As an implementation practitioner, **you are planning to implement the MOVE program within an acute care hospital to support a change in practice related to mobilisation of older adults**. To inform your work, you are looking to identify an appropriate knowledge translation theory, model or framework.

Scenario B:

Please take a moment to read the scenario aloud. Once you are familiar with the scenario, please take a few minutes to reflect on what you might want to consider, and complete the task described below using the support tool. As you are doing so, please "think aloud" and provide any feedback including saying what you are thinking, what you are looking at and what you are trying to do.

You will have up to 10 minutes to complete the task. Do you have any questions?

SCENARIO
Background: Adults aged 65 and older account for a high percentage of acute hospital stays. Evidence suggests that early and consistent mobilisation of older adults admitted to hospital can decrease acute care length of stay, increase functional status and increase rates of discharge to home. Yet, rates of mobilisation in patients admitted to hospitals remain low. To address this gap, the Mobilisation of Vulnerable Elders (MOVE) initiative aims to promote early and consistent mobilisation practices for older adults admitted to hospitals. The three key components of this evidence-based program are to: mobilise patients at least three times a day; use progressive, scaled mobilisation; and, assess mobility within 24 hours of the decision to admit.<br>Task: As an implementation practitioner, **you are planning to evaluate the implementation of the MOVE program across a network of acute care hospitals**. To inform your work, you are looking to identify an appropriate knowledge translation theory, model or framework.

Usability Study Interview Guide:

Read Informed Verbal Consent Script to participant, covering:
- *Welcome and introductions*
- *Purpose of research study and interview process*
- *Terms of consent*

** Start recording*
** Obtain consent and record time*

SCENARIO A (0) or B (1)

Great, let's begin! First, I am going to share the link to the tool in the chat.
**Share link to tool and ensure participant can successfully access it*

I have two scenarios for you, that will be presented in random order. You will have 10 minutes to complete each task and provide feedback on the tool while doing so. Here is your first scenario.
** Share screen (first scenario)*
** Provide up to 10 minutes for participant to complete the task and provide their feedback on the tool while doing so.*
** Start timer for 10 minutes*
** Ask participant to share their screen as they complete the task*

Notes on issues/feedback/suggestions during first task:

Great, thank you! Here is your second scenario. Again, you will have 10 minutes to complete the task and provide feedback on the tool while doing so.
** Share screen (second scenario)*
** Provide up to 10 minutes for participant to complete the task and provide their feedback on the tool while doing so.*
** Start timer for 10 minutes*
** Ask participant to share their screen as they complete the task*

Notes on issues/feedback/suggestions during second task:

SYSTEM USABILITY SCALE

I'm going to read out a series of 10 statements. On a scale of 1 to 5, 1 being strongly disagree and 5 being strongly agree, how would you rate each of the following statements?
** All items should be answered. If a participant feels that they cannot respond to a particular item, they should select the centre point of the scale (i.e., 3); Source: Brooke, 1995.*

- I think that I would like to use this tool frequently
- I found the tool unnecessarily complex
- I thought the tool was easy to use
- I think that I would need the support of a technical person to be able to use this tool
- I found the various functions in this tool were well integrated
- I thought there was too much inconsistency in this tool
- I would imagine that most people would learn to use this tool very quickly
- I found the tool very cumbersome to use
- I felt very confident using the tool
- I needed to learn a lot of things before I could get going with this tool

Questions on Tool Content

1. Are the questions in the tool clear and easy to understand? Why or why not?
    - Is there anything in this tool that you found difficult to understand?
    - Is the language appropriate?

2. Is there anything in this tool that you would change or remove? If so, what? Why?

3. Is there any information missing from this tool that you think would be helpful to include?

Questions on Tool Navigation & Format

4. Did you find the tool easy to use? Why or why not?

- Does the order of the questions make sense?
- Which aspects of the way the tool is organized do you like/not like?
- How would you like the information to be organized and displayed?

5. Do you find the tool visually appealing (e.g., layout, font, colours)? Why or why not?

Questions on Tool Purpose

6. Would you consider using a tool like this to inform your next implementation project? Why or why not?
   - Would this tool help you choose a knowledge translation theory, model or framework to inform your work? Why/why not?
   - Can you describe some advantages/disadvantages of using a tool like this?

Thank you for your feedback on the tool. We are coming to the end of the interview.

Other Questions

7. Before we wrap up, do you have any additional comments, concerns, or suggestions for improvements to share, regarding the tool?

8. Do you currently use any online tools to facilitate selecting a(n) implementation/knowledge translation theory, model or framework to inform your work?
   - If yes, which ones?
   - If no, why not?

Demographic Information

I have a few demographic questions to ask and then we will wrap up the interview.

9. For approximately how long (in years) have you been facilitating knowledge translation/implementation practice activities in a healthcare environment?

10. Could you please briefly describe, in a couple of words, the type of healthcare organization or environment in which you work? (e.g., rural or urban healthcare organization, teaching hospital, funding or regulatory organization, etc.)

Thank you so much for your time!

Appendix 4. Results of mapping exercise for 210 TMFs

Name of TMF	Taxonomy					KTA stage				Change level			Reference
	Process model	Evaluation framework	Determinant framework	Classic theory	Implementation theory	Select and adapt knowledge	Barriers/ facilitators and/ or implement strategies	Monitor knowledge use and/or evaluate outcomes	Sustain knowledge use	Individual	Organizational	System	
4E Framework for Knowledge Dissemination and Utilization			x				x			x	x	x	[1,2]
A Framework for Improvement			x				x			x			[3]
A Framework for Spread	x		x						x		x	x	[4]
A Model of Interpersonal Behavior				x			x			x			[5]
A Staged Model of Innovation Development and Diffusion of Health Promotion Programs	x					x	x	x	x	x	x	x	[6]
Academic Center for Evidence-Based Practice (ACE) Star Model of Knowledge Transformation	x					x	x	x		x	x		[7]
Action Research	x					x	x	x		x	x	x	[8-11]
Active Implementation Frameworks (AIF)	x		x			x	x	x	x	x	x		[12]
Adherence Model			x				x	x	x	x		x	[13]
Advancing Research and Clinical Practice through Close Collaboration (ARCC) Model of Evidence-Based Practice in Nursing and Healthcare	x		x				x	x		x	x	x	[14-15]
Affective Events Theory				x			x			x			[16]
An Action Theory Model of Consumption			x	x			x			x			[17]
An Organizational Theory of Innovation Implementation Effectiveness					x	x		x			x		[18]
Attitude, Social Influence and Self-efficacy (ASE) Model		x	x	x			x	x		x			[19-21]
Availability, Responsiveness Continuity (ARC): An	x					x		x		x	x	x	[22-24]

	Taxonomy					KTA stage				Change level			
Name of TMF	Process model	Evaluation framework	Determinant framework	Classic theory	Implementation theory	Select and adapt knowledge	Barriers/ facilitators and/ or implement strategies	Monitor knowledge use and/or evaluate outcomes	Sustain knowledge use	Individual	Organizational	System	Reference
Organizational & Community Intervention Model													
Behavioral-Ecological Model of AIDS Prevention			x				x			x			[25]
CAN-IMPLEMENT Framework	x					x	x	x	x	x	x	x	[26]
Capability Opportunity Motivation and Behaviour (COM-B)				x			x			x			[27]
CDC DHAP’s Research-to-Practice Framework	x					x	x	x			x	x	[28-29]
CIHR Model of Knowledge Translation	x					x	x	x		x	x	x	[30-32]
Classical Conditioning				x			x			x			[33]
Clinical Work Assessment Model	x				x		x				x		[34-35]
Cognitive Behavioural Theory				x			x			x			[36]
Cognitive-Behavioral Model of Relapse Prevention			x	x			x			x			[37]
Cognitive-Social Health Information-Processing (C-SHIP) Model				x			x			x			[38]
Co-KT Framework	x					x			x	x	x	x	[39]
CollaboraKTion Framework	x		x			x	x	x	x	x	x	x	[40]
Collaboration for Leadership in Applied Health Research and Care (CLAHRC) Approach to Implementation	x						x	x	x	x	x	x	[41]
Collaborative Model for Achieving Breakthrough Improvement	x					x	x	x			x		[42]
Collaborative Model for Knowledge Translation Between Research and Practice Settings	x					x	x			x	x	x	[43]

Name of TMF	Taxonomy					KTA stage				Change level			Reference
	Process model	Evaluation framework	Determinant framework	Classic theory	Implementation theory	Select and adapt knowledge	Barriers/ facilitators and/ or implement strategies	Monitor knowledge use and/or evaluate outcomes	Sustain knowledge use	Individual	Organizational	System	
Communication-Behavior Change Model	x					x	x	x		x			[44]
Community Coalition Action Theory (CCAT)			x				x		x		x	x	[45]
Community-Based Knowledge Translation Framework (or Applying Knowledge To Generate Action Framework)	x					x	x			x	x	x	[46]
Complex Innovations Implementation Framework			x			x	x		x	x	x		[47]
Conceptual Framework for Addressing Social Context of Health Behaviors			x				x	x		x	x		[48]
Conceptual Framework for Program Sustainability in Public Health			x						x		x	x	[49]
Conceptual Framework for Research Knowledge Transfer and Utilization	x		x			x	x	x			x		[50]
Conceptual Model by Lara et al.	x		x				x	x				x	[51]
Conceptual Model for the Diffusion of Innovations in Service Organizations			x				x			x	x	x	[52]
Conceptual Model of Implementation Research		x						x		x	x	x	[53]
Conceptual Model of Knowledge Utilization			x				x				x	x	[54]
Conduct and Utilization of Research in Nursing (CURN) Project Model	x					x	x	x	x	x	x	x	[55-58]
Consolidated Framework for Implementation Research (CFIR)			x				x				x	x	[59]
Continuous Quality Improvement (CQI) model	x							x		x	x	x	[60]

Name of TMF	Taxonomy					KTA stage				Change level			Reference
	Process model	Evaluation framework	Determinant framework	Classic theory	Implementation theory	Select and adapt knowledge	Barriers/ facilitators and/ or implement strategies	Monitor knowledge use and/or evaluate outcomes	Sustain knowledge use	Individual	Organizational	System	
Coordinated Implementation Model	x		x			x	x			x	x	x	[61]
Core Steps for Effective Implementation	x					x	x	x		x	x	x	[62]
Critical Realism & the Arts Research Utilization Model (CRARUM)	x		x				x	x		x	x		[63]
Davis' Pathman-PRECEDE Model	x						x		x	x	x	x	[64-66]
Design Focused Implementation Model	x						x	x		x	x	x	[67]
Development Strategy			x				x			x			[68]
Diffusion of Innovations				x			x		x	x	x	x	[69]
Dissemination of Evidence-Based Interventions to Prevent Obesity	x		x				x			x	x	x	[70]
Dynamic Sustainability Framework			x					x	x	x	x	x	[71]
E2D2 Model	x	x	x				x	x		x	x	x	[72]
Ecological Framework by Durlack & DuPre		x						x		x	x	x	[73]
Ecological Framework by Sallis & Owen			x				x			x	x	x	[74-75]
Ecological Model of Diabetes Prevention					x		x			x			[76]
Ecological Model of Health Behaviour			x				x			x	x	x	[77]
Evidence-Driven Community Health Improvement Process (EDCHIP)	x					x	x	x		x	x	x	[78]
Expectancy-Value Model				x			x			x			[79]
Exploration, Preparation, Implementation, Sustainment (EPIS) Framework	x		x			x	x		x		x	x	[80]

Name of TMF	Taxonomy					KTA stage				Change level			Reference
	Process model	Evaluation framework	Determinant framework	Classic theory	Implementation theory	Select and adapt knowledge	Barriers/ facilitators and/ or implement strategies	Monitor knowledge use and/or evaluate outcomes	Sustain knowledge use	Individual	Organizational	System	
Explore Values, Operationalize and Learn, and eValuate Efficacy (EVOLVE)	x						x	x		x	x	x	[81]
Extended Information Processing Model (or Information Processing Model of Attitude Change and its extension or McGuire's persuasion matrix)	x		x				x			x			[82]
Extended Parallel Process Model (EPPM)				x			x			x			[83]
Factors Determining Rate of Adoption of Research Innovations into Practice			x			x	x				x		[84]
Framework by Cochrane et al.		x	x				x			x	x	x	[85]
Framework by Ferlie and Shortell			x				x			x	x	x	[86]
Framework by Grol & Wensing	x		x				x			x	x	x	[87]
Framework by Gurses et al.		x	x				x	x		x			[88]
Framework For Analyzing Adoption of Complex Health Innovations		x					x			x	x		[89-90]
Framework for Conceptualizing Program Sustainability			x						x		x	x	[91]
Framework for Knowledge Translation (or Understanding-User-Context Framework)			x			x	x			x	x		[92]
Framework for the Dissemination & Utilization of Research for Health-Care Policy & Practice	x		x				x	x	x	x	x	x	[93]
Framework for the Transfer of Patient Safety Research into Practice	x					x	x	x			x	x	[94]

Name of TMF	Taxonomy					KTA stage				Change level			Reference
	Process model	Evaluation framework	Determinant framework	Classic theory	Implementation theory	Select and adapt knowledge	Barriers/ facilitators and/ or implement strategies	Monitor knowledge use and/or evaluate outcomes	Sustain knowledge use	Individual	Organizational	System	
Framework of Dissemination in Health Services Intervention Research	x						x	x	x	x	x	x	[95]
General Theory of Deviant Behaviour				x			x			x			[96]
Goal Directed Theory				x			x			x			[97]
Goal Framing Theory				x			x			x			[98]
Goal Setting Theory				x			x	x		x			[99]
Health Action Process Approach (HAPA)				x			x			x			[100]
Health Behavior Framework			x		x		x			x			[101]
Health Behaviour Goal Model			x				x			x			[102]
Health Behaviour Internalization Model			x				x			x			[103]
Health Belief Model			x	x			x			x			[104]
Health Communication Program Cycle	x					x	x	x		x	x	x	[105]
Health Promotion Model			x				x			x			[106]
Health Promotion Technology Transfer Process	x					x	x	x		x	x	x	[107]
Healthcare Improvement Collaborative Model (HICM)	x						x	x	x		x		[108]
Hierarchical Model of Intrinsic and Extrinsic Motivation			x	x			x			x			[109]
I-Change Model (or Integrated Change Model)			x				x			x			[110-111]
Implementation Effectiveness Model					x		x	x			x		[112-113]
Implementation Process Model	x					x	x	x	x	x	x		[114]
Information Technology Adoption Model (ITAM)		x	x				x	x		x			[115]
Information-Motivation-Behavioural (IMB) Skills Model of AIDS-Preventive Behavior				x			x			x			[116-118]
Institutional Theory				x			x			x			[119]

Name of TMF	Taxonomy					KTA stage				Change level			Reference
	Process model	Evaluation framework	Determinant framework	Classic theory	Implementation theory	Select and adapt knowledge	Barriers/ facilitators and/ or implement strategies	Monitor knowledge use and/or evaluate outcomes	Sustain knowledge use	Individual	Organizational	System	
Integrated Mobile Ecological Model for the Promotion of Physical Activity			x				x			x	x	x	[120]
Integrated Social-Cognitive Framework			x	x			x			x			[121]
Integrated Theoretical Model for Alcohol and Drug Prevention			x	x			x			x	x	x	[122]
Integrated Theory of Drinking and Behaviour			x	x			x			x	x	x	[123]
Integrating Factors Influencing Smoking Behaviour and The Model of Attitude and Behaviour Change			x	x			x			x			[124]
Integrative Factors Influencing Smoking Behaviour Model			x	x			x			x			[124]
Integrative Model of Behavioural Prediction			x				x			x	x	x	[125]
Integrative Model of Health and Attitude Behaviour Change				x			x			x			[124]
Integrative Theory of Health Behaviour Change			x	x			x			x			[126]
Interactive Systems Framework (ISF) for Dissemination and Implementation	x		x			x	x				x	x	[127]
Intervention Mapping Framework	x					x	x	x	x	x	x	x	[128-129]
Iowa Model of Evidence-based Practice to Promote Quality Care and revision	x					x		x	x	x	x		[130]
Knowledge Exchange Framework			x			x	x		x	x	x	x	[131]
Knowledge Exchange-Decision Support (KE-DS) Model	x		x			x	x	x		x	x	x	[132-133]

Name of TMF	Taxonomy					KTA stage				Change level			Reference
	Process model	Evaluation framework	Determinant framework	Classic theory	Implementation theory	Select and adapt knowledge	Barriers/ facilitators and/ or implement strategies	Monitor knowledge use and/or evaluate outcomes	Sustain knowledge use	Individual	Organizational	System	
Knowledge Translation Model of Tehran University of Medical Sciences	x		x			x	x			x	x		[134-135]
Knowledge-to-Action (KTA) Framework	x					x	x	x	x	x	x	x	[136]
KT Framework for AHRQ Patient Safety Portfolio and Grantees	x					x	x		x	x	x		[137]
Language Expectancy Theory				x			x			x			[138]
LEAN Methodology and Transformation Process	x							x			x		[139]
Lewin's Change Theory	x					x	x		x	x	x		[140]
Model by Davis et al.	x					x	x	x	x	x	x	x	[141]
Model by Huberman	x					x	x			x	x	x	[142]
Model by Landry et al. (or The Ladder of Knowledge Utilization)	x						x			x			[143]
Model for Improvement	x					x	x	x			x		[144]
Model of Community-Based Program Sustainability		x	x						x			x	[145]
Model of Pro-Environmental Behavior			x				x			x			[146]
Model of Research Utilization in Occupational Therapy	x					x	x			x			[147]
Motivation-Opportunities-Abilities (MOA) Model of Consumer Behavior			x	x			x			x			[148]
Multilevel Conceptual Framework of Organizational Innovation Adoption			x				x			x	x		[149]
National Center on Health, Physical Activity and Disability (NCHPAD) Knowledge, Adaptation, Translation and Scale-up (N-KATS)	x					x	x	x	x	x	x	x	[150]
Navigation Chart	x						x	x	x		x		[151]

Name of TMF	Taxonomy					KTA stage				Change level			Reference
	Process model	Evaluation framework	Determinant framework	Classic theory	Implementation theory	Select and adapt knowledge	Barriers/ facilitators and/ or implement strategies	Monitor knowledge use and/or evaluate outcomes	Sustain knowledge use	Individual	Organizational	System	
Needs-Opportunities-Abilities (NOA) Model of Consumer Behaviour			x				x			x			[152]
NHS Sustainability Model		x	x						x	x	x		[153]
Normalization Process Theory					x		x			x	x		[154-156]
Organizational Development Theory	x	x	x		x	x	x	x			x		[157-158]
Organizational Readiness for Change (ORC) Theory			x		x	x	x				x		[159]
Organizational Theory of Implementation Effectiveness			x		x		x	x			x		[160]
Ottawa Model of Research Use (OMRU)	x		x				x	x		x	x	x	[161-163]
Participatory Innovation Diffusion Model			x				x				x	x	[164]
Participatory Organization Development (OD) Framework for Scaling Up Quality-of-Care Innovations			x				x					x	[165]
Pathways to Evidence Informed Policy (or Evidence-Informed Policy and Practice Pathway)	x					x	x			x	x	x	[166]
PEN-3 Cultural Model			x				x			x			[167-168]
Plan-Do-Study-Act (PDSA) Cycles	x						x	x		x	x	x	[169]
Political Economy of Health				x			x			x	x	x	[170]
PPRNet-TRIP Quality Improvement Model	x						x				x		[171]
Practical Robust Implementation and Sustainability Model (PRISM)		x	x				x	x	x	x	x	x	[172]
Practice Change and Development Model			x				x			x	x		[173]
Precaution Adoption Process Model (PAPM)					x		x			x			[174]
PRECEDE-PROCEED Model	x						x	x		x	x	x	[175-176]

Name of TMF	Taxonomy					KTA stage				Change level			Reference
	Process model	Evaluation framework	Determinant framework	Classic theory	Implementation theory	Select and adapt knowledge	Barriers/ facilitators and/ or implement strategies	Monitor knowledge use and/or evaluate outcomes	Sustain knowledge use	Individual	Organizational	System	
PRIME Theory of Motivation				x			x			x			[177-178]
Priming Theory				x			x			x	x	x	[179]
Problem Behavior Theory		x	x				x	x		x			[180]
Problem Solving Model	x						x	x		x			[181]
Process Marker Model	x							x		x	x	x	[182]
Program-Planning Model	x					x	x	x		x			[183-184]
Promoting Action on Research Implementation in Health Services (PARIHS) Framework		x	x		x	x	x			x	x	x	[185-187]
Pronovost’s 4E’s Process Theory	x					x	x	x	x	x	x	x	[188]
Prospect Theory				x			x			x			[189-190]
Public Health Action Model for Cancer Survivorship			x				x			x	x	x	[191]
Push-Pull Capacity Model			x				x				x		[192-194]
Quality Implementation Framework	x					x	x	x		x	x	x	[195]
Quality Improvement Supply Chain Model	x					x	x			x	x	x	[196]
Reach Effectiveness Adoption Implementation Maintenance (RE-AIM)		x						x	x	x	x	x	[197]
Real-World Dissemination			x				x			x	x	x	[198-199]
Regulative Research Cycle	x					x	x	x		x	x	x	[200]
Regulatory Fit Theory				x			x			x			[201-202]
Replicating Effective Programs Plus Framework	x					x	x	x	x	x	x	x	[203]
Research and Policy in International Development (RAPID) Framework			x				x			x	x	x	[204-205]
Research Knowledge Infrastructure			x				x				x		[206]
Risk as Feelings Model				x			x			x			[207]
Self-Determination Theory				x			x			x			[208]
Self-Efficacy Theory				x			x			x			[209-211]
Self-Regulation Theory				x			x			x			[212]

Name of TMF	Taxonomy					KTA stage				Change level			Reference
	Process model	Evaluation framework	Determinant framework	Classic theory	Implementation theory	Select and adapt knowledge	Barriers/ facilitators and/ or implement strategies	Monitor knowledge use and/or evaluate outcomes	Sustain knowledge use	Individual	Organizational	System	
Sequential Model of Behavioral and Social Change (or The Seven Doors Social Marketing Approach)			x				x			x			[213]
Six Staged Model of Communication Effects	x						x			x			[214]
Social Action Theory			x	x			x			x	x		[215]
Social Change Theory				x			x			x	x	x	[216]
Social Cognitive Theory				x			x			x			[217-218]
Social Consensus Model of Health Education			x				x			x	x	x	[219]
Social Ecology			x	x			x			x	x	x	[220]
Social Ecology Model for Health Promotion	x		x				x	x		x	x	x	[221-223]
Social Influence Model of Virtual Community Participation			x	x			x			x			[224]
Social Learning Theory				x			x			x			[225]
Social Marketing Framework			x				x				x		[226-227]
Social Marketing Planning Model	x					x	x	x		x	x	x	[228-229]
Social Norms Theory				x			x			x		x	[230]
Social Problem-Solving Model for Health Behaviour Change			x				x			x			[231]
Soft Systems Theory/Methodology	x					x	x	x			x	x	[232-233]
Stage Theory of Organizational Change	x					x	x		x		x		[234-235]
Stages of Research and Evaluation	x					x	x	x	x	x	x	x	[236]
Stetler Model of Research Utilization	x					x	x	x		x	x		[237]
Sticky Knowledge	x		x			x	x		x		x		[238-239]
Structural-Ecological Model			x				x			x	x	x	[240]
Systems Model of Health Behaviour Change				x			x			x		x	[241]

Name of TMF	Taxonomy: Process model	Taxonomy: Evaluation framework	Taxonomy: Determinant framework	Taxonomy: Classic theory	Taxonomy: Implementation theory	KTA stage: Select and adapt knowledge	KTA stage: Barriers/ facilitators and/ or implement strategies	KTA stage: Monitor knowledge use and/or evaluate outcomes	KTA stage: Sustain knowledge use	Change level: Individual	Change level: Organizational	Change level: System	Reference
Technology Acceptance Model			x	x			x			x			[242]
Technology Adoption Criteria in Health (TEACH) Model (or Technology Adoption Curve and its Critical Success Factors)	x						x			x			[243-244]
Temporal Self-Regulation Theory				x			x			x			[245]
Ten Steps to Systems Thinking	x						x	x				x	[246]
The Black Dog Institute Integrated Model of Knowledge Translation Exchange	x					x	x	x	x	x	x	x	[247]
Theoretical Domains Framework			x				x			x	x		[248-249]
Theories of Change Evaluation Model	x	x						x		x	x	x	[250]
Theory of Meaningful Learning (including Model)	x			x			x			x			[251-252]
Theory of Normative Conduct				x			x			x			[253]
Theory of Normative Social Behaviour				x			x			x			[254]
Theory of Planned Behaviour				x			x			x			[255]
Theory of Reasoned Action				x			x			x			[256]
Theory of Research Utilization Enhancement for Occupational Therapists (TRUE-OT)				x			x			x			[257]
Theory of Triadic Influence				x			x			x			[258]
Transcontextual Model of Motivation			x	x			x			x			[259]
Transtheoretical Model of Behaviour Change				x			x			x			[260]
University of Hawai‘i Center for ‘Ohana Self-Management of Chronic Illnesses (COSMCI) Conceptual Framework			x				x	x		x			[261]
Utilization-Focused Surveillance Framework			x			x	x	x			x	x	[262]

Name of TMF	Taxonomy					KTA stage				Change level			Reference
	Process model	Evaluation framework	Determinant framework	Classic theory	Implementation theory	Select and adapt knowledge	Barriers/ facilitators and/ or implement strategies	Monitor knowledge use and/or evaluate outcomes	Sustain knowledge use	Individual	Organizational	System	
Value Belief Norm Theory			x	x			x			x		x	[263]
Western Australia Health Network Policy Development and Implementation Cycle	x					x	x	x				x	[264]
Yin's Routinization Framework	x		x		x		x		x	x	x	x	[265-266]
**Total**	**86**	**16**	**97**	**59**	**12**	**61**	**192**	**76**	**41**	**172**	**126**	**96**	

Appendix 5. References for 210 TMFs from mapping exercise

[1] Farkas M et al. Knowledge dissemination and utilization in gerontology: an organizing framework. Gerontologist 2003;43(S1):S47.

[2] Farkas M, Anthony WA. Bridging science to service: using Rehabilitation Research and Training Center program to ensure that research-based knowledge makes a difference. J Rehabil Res Dev 2007;44(6):879-92.

[3] Cabana MD et al. Why don't physicians follow clinical practice guidelines? A framework for improvement. JAMA 1999;282:1458-65.

[4] Massoud MR et al. A Framework for Spread: From Local Improvements to System-Wide Change. IHI Innovation Series white paper. Cambridge, MA: Institute for Healthcare Improvement; 2006. (Available on www.IHI.org).

[5] Triandis H. Interpersonal Behavior, Brooks/Cole Pub. Co, 1977.

[6] Oldenburg B et al. How does research contribute to evidence-based practice in health promotion? Health Promotion Journal of Australia 1996;6(2):15-20.

[7] Stevens KR. ACE Star Model of EBP: Knowledge Transformation. 2004; Available from: www.acestar.uthscsa.edu.

[8] Meyer JE. New paradigm research in practice: the trials and tribulations of action research. J Adv Nurs 1993;18(7):1066-72.

[9] Lewin K. Action research and minority problems. Journal of Social Issues 1946;2:34-46.

[10] Ferrance E. Action Research. Northeast and Islands Regional Educational Laboratory At Brown University, 2000.

[11] Leykum LK et al. Implementation research design: integrating participatory action research into randomized controlled trials. Implement Sci 2009;4:69.

[12] Fixsen DL et al. Implementation research: a synthesis of the literature. Tampa FL: University of South Florida, Louis de la Parte Florida Mental Health Institute, The National Implementation Research Network, 2005 (FMHI Publ. #231).

[13] Bastani R et al. Tailored risk notification for women with a family history of breast cancer. Prev Med. 1999;29(5):355-64.

[14] Melnyk BM, Fineout-Overholt E. Evidence-based practice in nursing and healthcare: A guide to best practice. Philadelphia: Lippincott, Williams and Wilkins; 2005, p257.

[15] Melnyk BM. Achieving a high-reliability organization through implementation of the ARCC model for systemwide sustainability of evidence-based practice. Nurs Adm Q 2012;36(2):127-35.

[16] Weiss, Cropanzano. Affective Events Theory: A Theoretical Discussion of The Structure, Cause and Consequences of Affective Experiences at Work. Research in Organizational Behavior 1996.

[17] Bagozzi RP. The Poverty of Economic Explanations of Consumption and an Action Theory Alternative. Managerial and Decision Economics 2000;21:95-109.

[18] Turner K et al. Testing the organizational theory of innovation implementation effectiveness in a community pharmacy medication management program: a hurdle regression analysis. Implement Sci 2018;13(1):105.

[19] van Es SM et al. Predicting adherence to prophylactic medication in adolescents with asthma: an application of the ASE-model. Patient Educ Couns 2002;47(2):165-71.

[20] de Vries H et al. Self-efficacy: third factor besides attitude and subjective norm as a predictor of behavioral intentions. Health Educ Res 1988;3:273–82.

[21] de Vries H et al. The impact of social influences in the context of attitude, self-efficacy, intention and previous behaviour as predictors of smoking onset. J Appl Soc Psychol 1995;25:237-57.

[22] Glisson C, Schoenwald SK. The ARC organizational and community intervention strategy for implementing evidence-based children's mental health treatments. Mental Health Serv Res 2005;7(4):243-59.

[23] Glisson C et al. Randomized trial of MST and ARC in a two-level evidence-based treatment implementation strategy. J Consult Clin Psychol 2010;78(4):537-50.

[24] Glisson C et al. The effects of the ARC organizational intervention on caseworker turnover, climate, and culture in children's service systems. Child Abuse Neglect 2006;30(8):855-80.

[25] Hovell MF et al. A Behavioral-Ecological Model of Adolescent Sexual Development: A Template for AIDS Prevention. The Journal of Sex Research 1994;31(4):267-281.
[26] Harrison MB et al. Guideline adaptation and implementation planning: a prospective observational study. Implement Sci 2013;8:49.
[27] Michie S et al. The behaviour change wheel: a new method for characterising and designing behaviour change interventions. Implement Sci 2011;6:42.
[28] Collins CB et al. A Comparison of the Interactive Systems Framework (ISF) for Dissemination and Implementation and the CDC Division of HIV/AIDS Prevention's Research-to-Practice Model for Behavioral Interventions. Am J Community Psychol 2012;50:518-29.
[29] Collins C et al. The diffusion of effective behavioral interventions project: Development, implementation, and lessons learned. AIDS Education and Prevention 2006;18(4 Suppl. A):5-20.
[30] Armstrong R et al. The role and theoretical evolution of knowledge translation and exchange in public health. J Public Health (Oxf) 2006;28:384-9.
[31] Brachaniec M et al. The Institute of Musculoskeletal Health and Arthritis (IMHA) knowledge exchange task force: An innovative approach to knowledge translation. JCCA J Can Chiropr Assoc 2006;50:8-13.
[32] Graham ID et al. Lost in knowledge translation: Time for a map? J Contin Educ Health Prof 2006;26:13-24.
[33] McLeod SA. Classical conditioning. 2018. Retrieved from https://www.simplypsychology.org/classical-conditioning.html.
[34] Sams L et al. The Challenge of Using Evidence-Based Practice. JONA 2004;34(9):407-414.
[35] Sams L, Gannon ME. Evidence-based practice and clinical work assessment. Semin Periop Nurs 2000;9(3):125-32.
[36] Beck A et al. Cognitive Therapy of Depression. New York: Guilford Press; 1979.
[37] Stuart RB, editor. Adherence, compliance and generalization in behavioral medicine. New York: Brunner/Mazel; 1982.
[38] Miller SM et al. Applying cognitive-social theory to health-protective behavior: Breast self-examination in cancer screening. Psychological Bulletin; Washington 1996;119(1):70.
[39] Kitson A et al. Knowledge translation within a population health study: how do you do it? Implement Sci 2013;8(1):54.
[40] Jenkins EK et al. Strengthening population health interventions: developing the CollaboraKTion Framework for Community-Based Knowledge Translation. Health research policy and systems 2016;14(1):65.
[41] Harvey G et al. The NIHR Collaborations for Leadership in Applied Health Research and Care (CLAHRC) for Greater Manchester: combining empirical, theoretical and experiential evidence to design and evaluate a large-scale implementation strategy. Implement Sci 2011;6:96.
[42] Institute for Healthcare Improvement. The Breakthrough Series: IHI's Collaborative Model for Achieving Breakthrough Improvement. Diabetes Spectrum 2004 Apr; 17(2): 97-101.
[43] Baumbusch JL et al. Pursuing common agendas: a collaborative model for knowledge translation between research and practice in clinical settings. Res Nurs Health 2008;31(2):130-40.
[44] McGuire W. Public communication as a strategy for inducing health-promoting behavior change. Prev Med 1984; 13:299-319.
[45] DiClemente RJ et al, editors. Emerging theories in health promotion practice and research. San Francisco (CA): Jossey-Bass; 2002.
[46] Campbell B. Applying knowledge to generate action: A community-based knowledge translation framework. J Contin Educ Health Prof 2010;30(1):65-71.
[47] Helfrich CD et al. Determinants of implementation effectiveness: adapting a framework for complex innovations. Med Care Res Rev 2007;64(3):279-303.
[48] Sorensen G. Model for incorporating social context in health behavior interventions: applications for cancer prevention for working-class, multiethnic populations. Prev Med 2003;37(3):188-97.
[49] Schell et al. Public health program capacity for sustainability: a new framework. Implementation Science 2013;8:15.
[50] Kramer DM, Cole DC. Sustained, intensive engagement to promote health and safety knowledge transfer to and utilization by workplaces. Sci Commun 2003;25(1):56.
[51] Lara M. Balancing "fidelity" and community context in the adaptation of asthma evidence-based interventions in the "real world". Health Promot Pract. 2011;12(6 Suppl 1):63S-72S.

[52] Greenhalgh T et al. Diffusion of innovations in service organizations: systematic review and recommendations. Milbank Q 2004;82(4):581-629.

[53] Proctor EK et al. Implementation research in mental health services: an emerging science with conceptual, methodological, and training challenges. Admin Policy Mental Health 2009;36(1):24-34.

[54] Lester JP. The utilization of policy analysis by state agency officials. Sci Commun 1993;14(3):267.

[55] Horsley J et al. Research utilization as an organizational process. JONA 1978;8(7):4-6.

[56] Brown GT, Rodger S. Research utilization models: Frameworks for implementing evidence-based occupational therapy practice. Occupational Therapy International 1999;6(1):1-23.

[57] Sparkman ED et al. Putting Research into Practice. Rehabilitation Nursing 1991;16(1):12-14.

[58] Horsley J. Using research to improve nursing practice: A guide. Orlando, FL: Grune & Stratton, Inc; 1983.

[59] Damschroder LJ et al. Fostering implementation of health services research findings into practice: a consolidated framework for advancing implementation science. Implement Sci 2009;4:50.

[60] U.S. Department of Health & Human Services Office of Adolescent Health. Continuous Quality Improvement (CQI) infosheet. https://www.hhs.gov/ash/oah/.

[61] Lomas J. Retailing research: increasing the role of evidence in clinical services for childbirth. Milbank Q 1993:439-75.

[62] Ilbawi AM, Anderson BO. Global cancer consortiums: moving from consensus to practice. Ann Surg Oncol 2015;22(3):719-27.

[63] Kontos PC, Poland BD. Mapping new theoretical and methodological terrain for knowledge translation: contributions from critical realism and the arts. Implement Sci 2009;4:1.

[64] Davis D et al. The case for knowledge translation: shortening the journey from evidence to effect. BMJ 2003;327(7405):33-5.

[65] Pathman DE et al. The awareness-to-adherence model of the steps to clinical guideline compliance: the case of pediatric vaccine recommendations. Medical Care 1996;34(9):873.

[66] Green LW, Kreuter MW. Health program planning: an educational and ecological approach. 4th ed. New York: McGraw-Hill; 2005.

[67] Ramaswamy R et al. Making complex interventions work in low resource settings: Developing and applying a design focused implementation approach to deliver mental health through primary care in India. International Journal of Mental Health Systems Vol 12 2018, ArtID 5. 2018;12.

[68] Ashford G, Patkar S. The positive path: using appreciative inquiry in rural Indian communities. Department of International Development, Bangalore. 2001. Available at http://www.iisd.org/pdf/2001/ai_the_positive_path.pdf.

[69] Rogers EM. Diffusion of innovations. 5th ed. New York: Free Press, 2003.

[70] Dreisinger ML et al. Contextual factors influencing readiness for dissemination of obesity prevention programs and policies. Health Educ Res 2012;27(2):292-306.

[71] Chambers DA et al. The dynamic sustainability framework: addressing the paradox of sustainment amid ongoing change. Implementation Science 2013;8:117.

[72] Petermann L, Petz G. The E2D2 Model: A Dynamic Approach to Cancer Prevention Interventions. Health Promotion Practice 2011;12(4):561-68.

[73] Durlak JA, DuPre EP. Implementation matters: a review of research on the influence of implementation on program outcomes and the factors affecting implementation. Am J Community Psychol 2008;41:327-50.

[74] Sallis JF et al. An Ecological Approach to Creating More Physically Active Communities. Annual Review of Public Health 2006;27:297-322.

[75] Glanz K et al, editors. Health Behavior and Health Education: Theory, Research, and Practice. (3rd ed.) San Francisco: Jossey-Bass, 2002.

[76] Burnet D et al. A practical model for preventing type 2 diabetes in minority youth. The Diabetes Educator 2002;28:779-95.

[77] McLeroy KR et al. An Ecological Perspective on Health Promotion Programs. Health Education Quarterly, 1988;15:351-77.

[78] Layde P et al. A Model to Translate Evidence-Based Interventions Into Community Practice. American Journal of Public Health 2012;102(4):617-24.

[79] Fishbein M, Ajzen I. Belief, Attitude, Intention, and Behavior: An Introduction to Theory and Research. Reading, MA: Addison-Wesley; 1975.

[80] Aarons GA et al. Advancing a conceptual model of evidence-based practice implementation in public service sectors. Admin Policy Mental Health 2011;38(1):4-23.

[81] Peterson JC et al. Translating basic behavioral and social science research to clinical application: the EVOLVE mixed methods approach. J Consult Clin Psychol 2013;81(2):217-30.
[82] Flay BR et al. Mass media in health promotion: an analysis using an extended information-processing model. Health Educ Q 1980;7(2):127-47.
[83] Witte K. Putting the fear back into fear appeals: The extended parallel process model. Communication Monographs 1992;59(4):329-49.
[84] Bradley EH et al. Translating research into practice: speeding the adoption of innovative health care programs. Issue Brief (Commonw Fund) 2004;(724):1-12.
[85] Cochrane LJ et al. Gaps between knowing and doing: understanding and assessing the barriers to optimal health care. J Contin Educ Heal Prof 2007;27:94-102.
[86] Ferlie E, Shortell SM. Improving the quality of health care in the United Kingdom and the United States: a framework for change. Milbank Q 2001;79:281-315.
[87] Grol R, Wensing M. What drives change? Barriers to and incentives for achieving evidence-based practice. Med J Aust 2004;180:S57-60.
[88] Gurses AP. Using an interdisciplinary approach to identify factors that affect clinicians' compliance with evidence-based guidelines. Crit Care Med 2010;38(8 Suppl):S282-91.
[89] Atun RA et al. Diffusion of complex health innovations—implementation of primary health care reforms in Bosnia and Herzegovina. Health Policy Plan 2007;22(1):28-39.
[90] Atun R et al. Integration of targeted health interventions into health systems: a conceptual framework for analysis. Health Policy Plan 2010;25(2):104-11.
[91] Shediac-Rizkallah MC, Bone LR. Planning for the sustainability of communitybased health programs: Conceptual frameworks and future directions for research, practice and policy. Health Education Research 1998;13(1):104.
[92] Jacobson N et al. Development of a framework for knowledge translation: understanding user context. J Health Serv Res Policy 2003;8(2):94-9.
[93] Dobbins M et al. A framework for the dissemination and utilization of research for health-care policy and practice. Online J Knowl Synth Nurs 2002;9:7.
[94] Nieva VF et al. From science to service: a framework for the transfer of patient safety. In: Advances in patient safety: from research to implementation. Vol. 2. AHRQ Publ 050021. Rockville, MD: Agency for Healthcare Research and Quality, 2005.
[95] Mendel P et al. Interventions in organizational and community context: a framework for building evidence on dissemination and implementation in health services research. Adm Policy Ment Health 2008;35(1–2):21-37.
[96] Kaplan HB, Lin CH. Deviant identity, negative self-feelings, and decreases in deviant behavior: the moderating influence of conventional social bonding. Psychology, Crime and Law 2005;11:289-303.
[97] Bagozzi RP. The Self-Regulation of Attitudes, Intentions, and Behavior. Social psychology quarterly 1992;55:178-204.
[98] van Trijp H, editor. Encouraging Sustainable Behavior. New York: Psychology Press; 2013.
[99] Lunenburg FC. Goal-Setting Theory of Motivation. International Journal of Management, Business, and Administration 2011;15(1).
[100] Schwarzer R et al. Mechanisms of health behavior change in persons with chronic illness or disability: the Health Action Process Approach (HAPA). Rehabil Psychol 2011;56(3):161-70.
[101] Bastani R et al. Integrating Theory into Community Interventions to Reduce Liver Cancer Disparities: The Health Behavior Framework. Prev Med 2010;50(1-2):63-7.
[102] Maes S, Gebhardt W. Self-regulation and health behavior: The health behavior goal model. In M. Boekaerts, P. R. Pintrich, & M. Zeidner (Eds.), Handbook of self-regulation (pp. 343-368). San Diego, CA, US: Academic Press; 2000.
[103] Bellg AJ. Maintenance of health behavior change in preventive cardiology. Internalization and self-regulation of new behaviors. Behavior Modification 2003;27:103-31.
[104] Rosenstock IM. Historical Origins of the Health Belief Model. 1974.
[105] Making Health Communication Programs Work. A Planner's Guide. 2002. National Institutes of Health (NIH) National Cancer Institute (NCI).
[106] Pender NJ. Health Promotion Model Manual. 2011
[107] Orlandi MA. Health promotion technology transfer: organizational perspectives. Can J Public Health 1996;87 (S2):S28–S33.

[108] Edward K-L et al. A multi-state, multi-site, multi-sector healthcare improvement model: implementing evidence for practice. Int J Qual Health Care 2017;29(5):740-4.
[109] Vallerand R. Toward a hierarchical model of intrinsic and extrinsic motivation. In M. P.Zanna (Ed.), Advances in experimental social psychology, Vol. 29 (pp. 271-360). New York: Academic Press; 1997.
[110] De Vries H et al. Self-efficacy: the third factor besides attitude and subjective norm as a predictor of behavioral intentions. Health Education Research 1988;3:273-82.
[111] De Vries H, Mudde A. Predicting stage transitions for smoking cessation applying the Attitude – Social influence – Efficacy Model. Psychology & Health 1998;13:369-85.
[112] Klein KJ et al. Implementing computerized technology: an organizational analysis. J Appl Psychol 2001;86(5):811.
[113] Klein KJ, Sorra JS. The challenge of innovation implementation. Acad Manag Rev 1996:1055-80.
[114] Brand C. Translating evidence into practice for people with osteoarthritis of the hip and knee. Clin Rheumatol 2007;26:1411-20.
[115] Dixon DR. The behavioral side of information technology. Int J Med Inform 1999;56(1–3):117-23.
[116] Fisher JD et al. Empirical tests of an information-motivation-behavioral skills model of AIDS-preventive behavior with gay men and heterosexual university students. Health Psychol 1994;13(3):238-50.
[117] Fisher JD, Fisher WA. Changing AIDS risk behavior. Psychological Bulletin 1992;111:455-74.
[118] Fisher JD et al. Effects of a conceptually-based AIDS risk reduction intervention on AIDS risk behavior change in university students. Manuscript in preparation, University of Connecticut, Department of Psychology, Storrs; 1993.
[119] Scott WR. Institutions and Organizations: Ideas and Interests. Los Angeles, CA: Sage Publications; 2008.
[120] Matsudo SM et al. Physical Activity Promotion: Experiences and Evaluation of the Agita Sao Paulo Program Using the Ecological Mobile Model. Journal of Physical Activity and Health 2004;1:81-97.
[121] Gucciardi DF et al. Understanding sport continuation: an integration of the theories of planned behaviour and basic psychological needs. Journal of Science and Medicine in Sport 2015.
[122] Gonzalez G. An integrated theoretical model for alcohol and other drug abuse prevention on the college campus Journal of College Student Development 1989;30:492-503.
[123] Wagenaar AC, Perry CL. Community Strategies for the Reduction of Youth Drinking: Theory and Application. 1994;4(2):319-45.
[124] Flay BR et al. Cigarette smoking: why young people do it and ways of preventing it. In McGrath P (ed), Pediatric and Adolescent Behavioural Medicine, New York Springer-Verlag; 1983.
[125] Fishbein M, Ajzen I. Ch 1 Introduction, from Predicting and changing behavior. New York, NY: Psychology Press; 2010.
[126] Ryan P. Integrated Theory of Health Behavior Change: background and intervention development. Clinical nurse specialist CNS 2009;23:161-70.
[127] Wandersman A et al. Bridging the gap between prevention research and practice: the interactive systems framework for dissemination and implementation. Am J Community Psychol 2008;41(3-4):171-81.
[128] Bartholomew LK et al. Intervention Mapping: A process for developing theory- and evidence based health education programs. Health Education and Behavior 1998;25(5):545-63.
[129] Bartholomew E et al. Planning health promotion programs: An Intervention Mapping approach (4th ed.). Hoboken, NJ: Wiley; 2016.
[130] Titler MG et al. The Iowa model of evidence-based practice to promote quality care. Crit Care Nurs Clin North Am 2001;13:497-509.
[131] Ward V et al. Exploring knowledge exchange: a useful framework for practice and policy. Soc Sci Med 2012;74(3);297-304.
[132] Kazanjian A et al. A structured approach to knowledge exchange: understanding the implementation of a cancer survivor program. Eur J Oncol Nurs 2012;16(4):399-405.
[133] Howard AF et al. The Knowledge Exchange-Decision Support Model: application to cancer navigation programs. Support Care Cancer 2014;22(2):367-74.
[134] Gholami J et al. How should we assess knowledge translation in research organizations; designing a knowledge translation self-assessment tool for research institutes (SATORI). Health Res Policy Syst 2011;9:10.
[135] Majdzadeh R et al. Knowledge translation for research utilization: design of a knowledge translation model at Tehran University of Medical Sciences. J Contin Educ Health Prof 2008;28(4):270-7.
[136] Graham ID et al. Lost in Knowledge Translation: Time for a Map? The Journal of Continuing Education in the Health Professions. 2006;26:13-24.

[137] Nieva VF MR et al. From Science to Service: A Framework for the Transfer of Patient Safety Research into Practice. United States: In: Henriksen K, Battles JB, Marks ES, et al., editors. Advances in Patient Safety: From Research to Implementation (Volume 2: Concepts and Methodology). Rockville (MD): Agency for Healthcare Research and Quality 2005.
[138] Burgoon et al. Ch7 Language Expectancy Theory, in Dillard JP, Pfau MW. The Persuasion Handbook: Developments in Theory and Practice (1st ed.). Thousand Oaks, CA: SAGE;2002.
[139] Lean. Available at: https://www.lean.org/WhatsLean/.
[140] Lewin K. Frontiers in Group Dynamics: Concept, Method and Reality in Social Science; Social Equilibria and Social Change 1947;1(1);5-41.
[141] Davis SM et al. Introduction and conceptual model for utilization of prevention research. Am J Prev Med 2007;33:1S.
[142] Huberman M. Research utilization: the state of the art. Knowledge and Policy 1994;7(4):13-33.
[143] Landry R et al. Climbing the ladder of research utilization: evidence from social science. Sci Commun 2001;22:396-422.
[144] National Health Service. Quality, Service Improvement and Redesign Tools: Improvement. Plan, Do, Study, Act (PDSA) cycles and the model for improvement. Available at https://aqua.nhs.uk/wp-content/uploads/2023/07/qsir-pdsa-cycles-model-for-improvement.pdf
[145] Mancini JA, Marek LL. Sustaining community-based programs for families: Conceptualization and measurement. Family Relations 2004;53(4):339-47.
[146] Kollmuss A, Agyeman J. Mind the Gap: Why do people act environmentally and what are the barriers to pro-environmental behavior? Environmental Education Research 2002;8(3):239-60.
[147] Craik J, Rappolt S. Theory of research utilization enhancement: a model for occupational therapy. Can J Occup Ther 2003;70(5):266-75.
[148] Olander F, Thogersen J. Understanding consumer behavior as prerequisite for environmental protection. Journal of Consumer Policy 1995;18:345-85.
[149] Frambach RT, Schillewaert N. Organizational innovation adoption: a multi-level framework of determinants and opportunities for future research. J Bus Res 2002;55(2):163-76.
[150] Rimmer JH et al. A New Framework and Practice Center for Adapting, Translating, and Scaling Evidence-Based Health/Wellness Programs for People With Disabilities. J Neurol Phys Ther 2016;40(2):107-14.
[151] Kubina N, Kelly J. Navigating self management. Australian Government Department of Health and Ageing.
[152] Gatersleben B, Vlek C. Household Consumption, Quality of Life and Environmental Impacts. In, K.J. Noorman, A.J.M. Schoot-Uiterkamp (Eds.), Green Households? Domestic Consumers, Environment and Sustainability, Earthscan, London; 1998, p141-83.
[153] National Health Service Institute for Innovation and Improvement. Sustainability Model and Guide, 2010.
[154] May C, Finch T. Implementing, embedding, and integrating practices: an outline of normalization process theory. Sociology: J Brit Sociol Assoc 2009;43(3):535-54.
[155] May CR et al. Development of a theory of implementation and integration: Normalization Process Theory Implement Sci 2009;4:29.
[156] Murray E et al. Normalisation process theory: a framework for developing, evaluating and implementing complex interventions. BMC Med 2010;8:63.
[157] Glanz et al. Health Behavior and Health Education. Theory, Research, and Practice. 4th Ed. San Fransisco, USA: Wiley, 2008.
[158] Cummings T. Organizational Development and Change. In J. Boonstra (ed.), Dynamics of Organizational Change and Learning. West Sussex, England: Wiley, 2004.
[159] Weiner BJ. A theory of organizational readiness for change. Implement Sci 2009;4:67.
[160] Weiner BJ et al. Using organization theory to understand the determinants of effective implementation of worksite health promotion programs. Health Educ Res 2009;24(2):292-305.
[161] Graham ID, Logan J. Innovations in knowledge transfer and continuity of care. Can J Nurs Res 2004;36(2):89-103.
[162] Logan J, Graham ID. Toward a comprehensive interdisciplinary model of health care research use. Sci Commun 1998;20(2):227.
[163] Logan J, Graham ID. The Ottawa Model of Research Use. In Bucknall JR-MaT ed. Models and frameworks for implementing evidence-based practice: evidence to action. Oxford: Wiley-Blackwell, 2010.

[164] Abed J et al. Comprehensive cancer control initiative of the Centers for Disease Control and Prevention: an example of participatory innovation diffusion. J Public Health Manag Pract 2000;6(2):79-92.
[165] Simmons R et al. Facilitating large-scale transitions to quality of care: an idea whose time has come. Stud Fam Plann 2002;33(1):61-75.
[166] Bowen S, Zwi AB. Pathways to "evidence-informed" policy and practice: a framework for action. PLoS Med 2005;2(7):e166.
[167] Airhihenbuwa CO. Health and Culture: Beyond the Western paradigm. Thousand Oaks, CA: Sage; 1995.
[168] Iwelunmor J et al. Child malaria treatment decisions by mothers of children less than five years of age attending an outpatient clinic in south-west Nigeria: an application of the PEN-3 cultural model. Malar J 2010;9:354.
[169] Langley GJ et al. The Improvement Guide: A Practical Approach to Enhancing Organizational Performance (2nd ed). San Francisco, CA, Jossey-Bass, 2009.
[170] Minkler M et al. The political economy of health: a useful theoretical tool for health education practice. Int Q Community Health Educ 1994;15(2):111-26.
[171] Nemeth LS et al. Implementing change in primary care practices using electronic medical records: a conceptual framework. Implement Sci 2008;3:3.
[172] Feldstein AC, Glasgow RE. A practical, robust implementation and sustainability model (PRISM) for integrating research findings into practice. Jt Comm J Qual Patient Saf 2008;34(4):228-43.
[173] Cohen D et al. A practice change model for quality improvement in primary care practice. J Healthc Manag 2004;49(3):155-70.
[174] Weinstein ND et al. The Precaution Adoption Process Model, in Glanz K et al (eds.), Health Behavior and Health Education (4th ed), p123-47. San Francisco: Jossey-Bass, 2008.
[175] Crosby R, Noar SM. What is a planning model? An introduction to PRECEDE-PROCEED. J Public Health Dent. 2011;71 Suppl 1:S7-15.
[176] Green LW, Kreuter MW. Health program planning: an educational and ecological approach. 4th ed. New York: McGraw-Hill, 2005.
[177] Prime Theory. Available at: http://www.primetheory.com/index.php.
[178] McEwen A, West R. The PRIME approach to giving up smoking. Practice Nursing 2010;21(3):145-53.
[179] Goidel RK et al. Priming Theory and Ras Models: Toward an Integrated Perspective of Media Influence. American Politics Quarterly; Beverly Hills, Calif 1997;25(3):287.
[180] Jessor R. Risk behavior in adolescence: a psychosocial framework for understanding and action. J. Adolesc Health 1991;12(8):597-605.
[181] Crane J. Using Research in Practice: Research Utilization: Theoretical Perspectives. Western Journal of Nursing Research 1985;7(2):261-8
[182] Trochim W et al. Evaluating Translational Research: A Process Marker Model. Clin Transl Sci 2011;4(3): 153-62.
[183] Kreuter M et al. Tailoring health messages: Customizing communication with computer technology. Mahwah, New Jersey: Lawrence Erlbaum Associates; 2000.
[184] Short et al. Theory-and evidence-based development and process evaluation of the Move More for Life program: a tailored-print intervention designed to promote physical activity among post-treatment breast cancer survivors International Journal of Behavioral Nutrition and Physical Activity 2013;10:124.
[185] Kitson AJ et al. Evaluating the successful implementation of evidence into practice using the PARIHS framework: theoretical and practical challenges. Implementation Science 2008;3(1).
[186] Kitson A et al. Enabling the implementation of evidence based practice: a conceptual framework. Qual Health Care 1998;7(3):149-58.
[187] Rycroft-Malone J. Promoting action on research implementation in health services (PARIHS). In: Rycroft-Malone J, Bucknall T, editors. Models and frameworks for implementing evidence-based practice: linking evidence to action. Oxford: Wiley-Blackwell; 2010. p109-36.
[188] Pronovost PJ et al. Translating evidence into practice: a model for large scale knowledge translation. BMJ 2008;337.
[189] Tversky A, Kahneman D. The framing of decisions and the psychology of choice. Science 1981;211:453-8.
[190] Kahneman D, Tversky A. The Psychology of Preferences. Scientific American 1982;247:160-73.
[191] Moore AR et al. Public Health Action Model for Cancer Survivorship. Am J Prev Med 2015;49(6 Suppl 5):S470-6.

[192] Green LW et al. Inferring strategies for disseminating physical activity policies, programs, and practices from the successes of tobacco control. Am J Prev Med 2006;31(4S):S66-S81.
[193] Kerner J et al. Introduction to the special section on dissemination: Dissemination research and research dissemination: How can we close the gap? Health Psychol 2005;24:443-6.
[194] Kerner JF et al. Translating research into improved outcomes in comprehensive cancer control. Cancer Causes and Control 2005;16:27-40.
[195] Meyers DC et al. The quality implementation framework: a synthesis of critical steps in the implementation process. Am J Community Psychol 2012;50(3-4):462-80.
[196] McNeill D, Kelley E. How the national healthcare quality and disparities reports can catalyze quality improvement. Med Care 2005;43(3 Suppl):I82-8.
[197] Glasgow RE et al. Evaluating the public health impact of health promotion interventions: the RE-AIM framework. Am J Publ Health 1999;89(9):1322-7.
[198] Chambers D et al (adapted by). Leading clinical practice change. In: Dopson S, Mark AL, eds. Leading health care organizations. Houndmills, UK: Palgrave Macmillan, 2003.
[199] Pettigrew AM et al. Shaping strategic change: making change in large organizations: the case of the National Health Service. Thousand Oaks CA: Sage, 1992.
[200] van Strien PJ. Towards of methodology of psychological practice, the regulative cycle. Theory & Psychology 1997;7(5):684-700.
[201] Higgins ET. Making a good decision: Value from fit. American Psychologist 2000;55:1217-30.
[202] Higgins ET. Value From Regulatory Fit. Current Directions in Psychological Science 2005;14(4):209-13.
[203] Kilbourne AM et al. Implementing evidence-based interventions in health care: application of the replicating effective programs framework. Implemen Sci 2007;2:42.
[204] Crewe E, Young J. Bridging Research and Policy: Context, Evidence and Links, ODI Working Paper No 173, ODI, London, 2002. Available at www.odi.org.
[205] RAPID Briefing Paper. Bridging Research and Policy in International Development, ODI.
[206] Ellen ME et al. Determining research knowledge infrastructure for healthcare systems: a qualitative study. Implement Sci 2011;6(1):60.
[207] Loewenstein GF et al. Risk as feelings. Psychological Bulletin 2001;127(2):267-86.
[208] Deci EL, Ryan RM. Self-Determination Theory and the Facilitation of Intrinsic Motivation, Social Development, and Well-Being. American Psychologist 2000;55(1):68-78.
[209] Bandura A, Adams NE. An Analysis of Self-Efficacy Theory of Behavior Change. Cognitive Therapy and Research 1977;1:125-39.
[210] Bandura A. Self-efficacy: Toward a unifying theory of behavioral change. Psychology Review 1977;84:191-215.
[211] Bandura A. Self-Efficacy Mechanism in Human Agency. American Psychologist 1982;37:22-47.
[212] Baumeister R et al. Self-Regulation and the Executive Function: The Self as Controlling Agent. In: Kruglanski AW, Higgins ET, editors. Social psychology: Handbook of basic principles. 2nd ed. New York: Guilford; 2011.
[213] Robinson L. The Seven Doors Social Marketing Approach. 1998. Available at https://www.comminit.com/en/node/201090.
[214] Vaughan PW, Rogers EM. A staged model of communication effects: Evidence from an entertainment-education radio soap opera in Tanzania. Journal of Health Communication 2000;5:203-27.
[215] Ewart CK. Social-Action Theory for a Public-Health Psychology. American Psychologist 1991;46:931-46.
[216] Thompson B, Kinne L. Social Change Theory: Applications to Community Health in, Health Promotion at the Community Level: New Advance. Ed Bracht N. Sage: 1999.
[217] McAlister AL et al. How individuals, environments, and health behaviors interact: Social Cognitive Theory, in Glanz et al. Health Behavior and Health Education. Theory, Research, and Practice. 4th Ed. San Fransisco, USA: Wiley, 2008. p169.
[218] Bandura A. Social Foundations of Thought and Action: A Social-Cognitive Theory. Prentice-Hall: Englewood Cliffs, NJ, 1986.
[219] Romer D, Hornik R. HIV education for youth: the importance of social consensus in behaviour change. AIDS Care 1992;4:285-303.
[220] Moos RH. Social-Ecological Perspectives on Health. In G. C. Stone, F. Cohen, and N. E. Adler (eds.), Health Psychology: A Handbook. San Francisco: Jossey-Bass, 1980.
[221] Stokols D. Establishing and Maintaining Healthy Environments: Toward a Social Ecology of Health Promotion. American Psychologist 1992;47:6-22.

[222] Stokols D et al. Increasing the Health Promotive Capacity of Human Environments. American Journal of Health Promotion 2003;18:4-13.
[223] Stokols D. Translating social ecological theory into guidelines for community health promotion. Am J Health Promot 1996;10(4):282-98.
[224] Dholakia UM et al. A social influence model of consumer participation in network- and small-group-based virtual communities. International Journal of Research in Marketing 2004;21:241-63.
[225] Bandura A. Social learning theory. General Learning Press; New York: 1977.
[226] Pirani S, Reizes T. The Turning Point Social Marketing National Excellence Collaborative: Integrating Social Marketing Into Routine Public Health Practice. Journal of Public Health Management & Practice 2005;11:131-8.
[227] Social marketing and public health: lessons from the field. A guide to social marketing from the Social Marketing National Excellence Collaborative. Seattle, WA: Turning Point National Program Office; 2003. Updated Feb 10, 2006; Available at https://www.sswm.info/node/1984.
[228] Kotier P, Zaltman G. Social marketing: an approach to planned social change. Journal of Marketing 1971;35:3-12.
[229] Kotler P. Social marketing of health behavior, in Frederiksen L et al (eds.), Marketing of health behavior: Principles, techniques and applications. New York: Plenum, 1983.
[230] Perkins W, Berkowitz AD. Perceiving the Community Norms of Alcohol Use among Students: Some Research Implications for Campus Alcohol Education Programming. International Journal of Addictions 1986;21:9-10, 961-976.
[231] Ewart CK. A social problem-solving approach to behavior change in coronary heart disease, in Shumaker SA et al (eds.), The handbook of health behavior change (p153-190). New York, NY, US: Springer Publishing Co, 1990.
[232] Gasson S. The Use of Soft Systems Methodology (SSM) As A Tool For Investigation. Warwick Business School 1994;1-18.
[233] Checkland P, Scholes J. Soft Systems Methodology in Action. Chichester, England: Wiley, 1990.
[234] Dunn Butterfoss F. Mobilizing organizations for health Promotion: Theories of organizational change, in Glanz et al. Health Behavior and Health Education. Theory, Research, and Practice. 4th Ed. San Fransisco, USA: Wiley, 2008. p338.
[235] Beyer JM, Trice HM. Implementing Change: Alcoholism Policies in Work Organizations. New York: Free Press, 1978.
[236] Nutbeam D, Bauman AE. Evaluation in a Nutshell: A Practical Guide to the Evaluation of Health Promotion Programs: McGraw-Hill; 2006.
[237] Stetler SB. Updating the Stetler Model of Research Utilization to Facilitate Evidence-Based Practice. Nurs Outlook 2001;49:272-9.
[238] Elwyn G et al. Sticky knowledge: a possible model for investigating implementation in healthcare contexts. Implement Sci 2007;2:44.
[239] Szulanski G. Exploring internal stickiness: impediments to the transfer of best practice within the firm. Strat Manag J 1996;17:27-43.
[240] Cohen DA et al. A Structural Model of Health Behavior: A Pragmatic Approach to Explain and Influence Health Behaviors at the Populations Level. Preventive Medicine 2000;30:146-54.
[241] Kersell MW, Milsum JH. A Systems Model of Health Behavior Change. Behavioral Science 1985;30(3): 119.
[242] Venkatesh V, Bala H. Technology Acceptance Model 3 and a Research Agenda on Interventions. Decision Sciences 2008;39:273-315.
[243] Leonard KJ. Critical Success Factors Relating to Healthcare's Adoption of New Technology: A Guide to Increasing the Likelihood of Successful Implementation. Healthcare Quarterly 2004;7(2):72-81.
[244] Leonard KJ, Dalziel S. How and when eHealth is a good investment for patients managing chronic disease. Healthc Manage Forum 2011;24(3):122-36.
[245] Hall PF. GT: Temporal self-regulation theory: A model for individual health behavior. Health Psychology Review 2007;1:6-52.
[246] WHO. 2009. Systems thinking for health systems strengthening. Edited by Don de Savigny and Taghreed Adam.
[247] Werner-Seidler A et al. An Australian example of translating psychological research into practice and policy: Where we are and where we need to go. Frontiers in Psychology 2016;7.

[248] Michie S et al, on behalf of the Psychological Theory Group. Making psychological theory useful for implementing evidence based practice: a consensus approach. Qual Saf Health Care 2005;14:26-33.
[249] Cane J et al. Validation of the theoretical domains framework for use in behaviour change and implementation research. Implement Sci 2012;7:37.
[250] Connel J, Kubisch A. Applying a theories of change approach to the evaluation of comprehensive community initiatives. New York City: Aspen Institute, 1996.
[251] Ausubel DP. The psychology of meaningful verbal learning. New York: Grune & Stratton; 1963.
[252] Ausubel DP. The acquisition and retention of knowledge: a cognitive view. Kluwer Academic Publishers, 2000.
[253] Cialdini RB et al. A Focus Theory of Normative Conduct - a Theoretical Refinement and Reevaluation of the Role of Norms in Human-Behavior. Advances in Experimental Social Psychology 1991;24:201-34.
[254] Rimal RN, Real K. How behaviors are influenced by perceived norms - A test of the theory of normative social behavior. Communication Research 2005;32:389-414.
[255] Ajzen I. The theory of planned behavior. Organizational Behavior and Human Decision Processes 1991;50:179-211.
[256] Ajzen I, Fishbein M. Understanding attitudes and predicting social behavior. Englewood Cliffs, NJ: Prentice-Hall, 1980.
[257] Craik J, Rappolt S. Theory of research utilization enhancement: a model for occupational therapy. Can J Occup Ther 2003;70(5):266-75.
[258] Flay et al. The Theory of Triadic Influence: A New Theory of Health Behavior With Implications for Preventive Interventions. Advances in Medical Sociology 1994;4:19-44.
[259] Hagger MS, Chatzisarantis NLD. The trans-contextual model of motivation. In Hagger MS, Chatzisarantis NLD (eds.), Intrinsic motivation and self-determination in exercise and sport (p53-70,309-313). Champaign, IL, US: Human Kinetics, 2007.
[260] Prochaska JO et al. Ch5 Transtheoretical model of behaviour change, in Glanz et al. Health Behavior and Health Education. Theory, Research, and Practice. 4th Ed. p97. San Fransisco, USA: Wiley, 2008
[261] Inouye J et al. A center for self-management of chronic illnesses in diverse groups. Hawaii Med J 2011;70(1):4-8.
[262] Green LW. Diffusion theory and knowledge dissemination, utilization, and integration in public health. Annu Rev Public Health 2009;30:151-74.
[263] Stern PC et al. A Value-Belief-Norm Theory of Support for Social Movements: The Case of Environmentalism. Huxley College on the Peninsulas Publications, 1999.
[264] Briggs AM et al. Applying a Health Network approach to translate evidence-informed policy into practice: A review and case study on musculoskeletal health. BMC Health Services Research 2012;12:394.
[265] Yin RK et al. Changing Urban Bureaucracies: How New Practices Become Routinized. 1978, RAND Corporation, Santa Monica, CA. Available at http://www.rand.org/pubs/reports/R2277.
[266] Yin RK. Life Histories of Innovations: How New Practices Become Routinized. Public Administration Review 1981;41(1):21-8.

Appendix 6. Screenshots of final tool homepage and results page

Screenshot of tool homepage

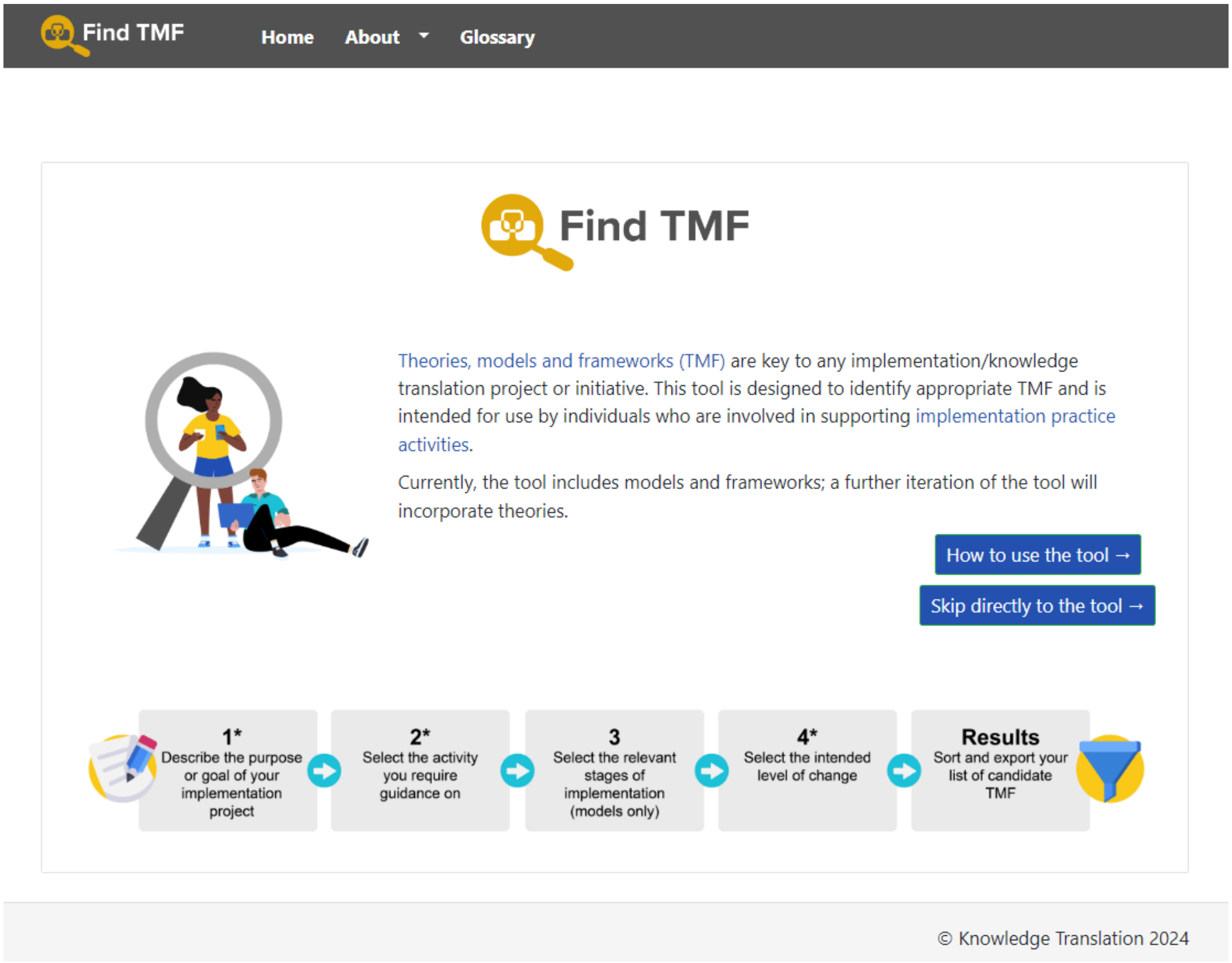

Screenshot of tool results page

Find TMF   Home   About   Glossary

1*	2*	3	4*	Results
Describe the purpose or goal of your implementation project	Select the activity you require guidance on	Select the relevant stages of implementation (models only)	Select the intended level of change	Sort and export your list of candidate TMF

Below is a summary of your answers followed by a list of TMF that may be applicable to your project.

Summary of answers:

1. You described the purpose or goal of your implementation project as:
To evaluate the implementation of the MOVE program across a network of acute care hospitals

2. You are looking for guidance on:
Understanding or explaining implementation outcomes and/or sustainability (i.e., an evaluation framework)

3. If you selected model in Q2, the relevant stages of implementation are:
n/a

4. The intended level of change is:
Individual, Organisational

TMF that may be applicable to your project:

Additional information is provided in the table for each TMF, to help you narrow down your list.

To sort your results alphabetically, click on a column heading.
To export your results, click on "Export" in the top right-hand corner of the table.

Complementary tools are listed here to help you make your final TMF selection.

Showing **1-10** of **10** items.   Export

Name of TMF	Original Publication	Year	PubMed Link	Citations	Model or Framework	Level of Change	Original Discipline or Condition	Figure with Included Concepts or Steps
E2D2 Model	Petermann L, Petz G. The E2D2 model: a dynamic approach to cancer prevention interventions. Health Promot Pract 2011;12(4):561-8.	2011	PubMed Article	2	Model & Evaluation Framework	All levels	Cancer, Health Promotion	Yes
Ecological Framework by Durlack & DuPre	Durlak JA, DuPre EP. Implementation matters: a review of research on the influence of implementation on program outcomes and the factors affecting implementation. Am J Community Psychol 2008;41:327-50.	2008	PubMed Article	3.6k	Evaluation Framework	All levels	Health Promotion	Yes
